# Significance of Shape Factor in Heat Transfer Performance of Molybdenum-Disulfide Nanofluid in Multiple Flow Situations; A Comparative Fractional Study

**DOI:** 10.3390/molecules26123711

**Published:** 2021-06-18

**Authors:** Asifa, Talha Anwar, Poom Kumam, Zahir Shah, Kanokwan Sitthithakerngkiet

**Affiliations:** 1 Department of Mathematics, Faculty of Science, King Mongkut’s University of Technology Thonburi (KMUTT), 126 Pracha-Uthit Road, Bang Mod, Thung Khru, Bangkok 10140, Thailand; asifaasifa1992@gmail.com (A.); anwartalha80@gmail.com (T.A.); 2 KMUTT Fixed Point Research Laboratory, SCL 802 Fixed Point Laboratory, Science Laboratory Building, Department of Mathematics, Faculty of Science, King Mongkut’s University of Technology Thonburi (KMUTT), Bangkok 10140, Thailand; 3 Department of Medical Research, China Medical University Hospital, China Medical University, Taichung 40402, Taiwan; 4 Center of Excellence in Theoretical and Computational Science (TaCS-CoE), Faculty of Science, King Mongkut’s University of Technology Thonburi (KMUTT), 126 Pracha-Uthit Road, Bang Mod, Thung Khru, Bangkok 10140, Thailand; 5 Department of Mathematical Sciences, University of Lakki Marwat, Lakki Marwat 28420, Khyber Pakhtunkhwa, Pakistan; zahir@ulm.edu.pk; 6 Intelligent and Nonlinear Dynamic Innovations Research Center, Department of Mathematics, Faculty of Applied Science, King Mongkut’s University of Technology North Bangkok (KMUTNB), 1518 Wongsawang, Bangsue, Bangkok 10800, Thailand; kanokwan.s@sci.kmutnb.ac.th

**Keywords:** comparative fractional study, shape factor, time-dependent conditions, laplace transform, heat transfer

## Abstract

In this modern era, nanofluids are considered one of the advanced kinds of heat transferring fluids due to their enhanced thermal features. The present study is conducted to investigate that how the suspension of molybdenum-disulfide $\left(MoS_{2}\right)$ nanoparticles boosts the thermal performance of a Casson-type fluid. Sodium alginate (NaAlg) based nanofluid is contained inside a vertical channel of width *d* and it exhibits a flow due to the movement of the left wall. The walls are nested in a permeable medium, and a uniform magnetic field and radiation flux are also involved in determining flow patterns and thermal behavior of the nanofluid. Depending on velocity boundary conditions, the flow phenomenon is examined for three different situations. To evaluate the influence of shape factor, $MoS_{2}$ nanoparticles of blade, cylinder, platelet, and brick shapes are considered. The mathematical modeling is performed in the form of non-integer order operators, and a double fractional analysis is carried out by separately solving Caputo-Fabrizio and Atangana-Baleanu operators based fractional models. The system of coupled PDEs is converted to ODEs by operating the Laplace transformation, and Zakian’s algorithm is applied to approximate the Laplace inversion numerically. The solutions of flow and energy equations are presented in terms of graphical illustrations and tables to discuss important physical aspects of the observed problem. Moreover, a detailed inspection on shear stress and Nusselt number is carried out to get a deep insight into skin friction and heat transfer mechanisms. It is analyzed that the suspension of $MoS_{2}$ nanoparticles leads to ameliorating the heat transfer rate up to 9.5%. To serve the purpose of achieving maximum heat transfer rate and reduced skin friction, the Atangana-Baleanu operator based fractional model is more effective. Furthermore, it is perceived that velocity and energy functions of the nanofluid exhibit significant variations because of the different shapes of nanoparticles.

## 1. Introduction

Nanotechnology, described as the development of atomic-scale structures through appropriate manipulation of molecules and atoms, is influencing a wide variety of modern physical phenomena in today’s world. For example, personal computers are more advanced, faster, less expensive, and hold enhanced storage capacities. Silver nanoparticles are coated on bandages for the speedy healing of the cuts and wounds. Nanomaterials are being utilized in the manufacturing of automobiles, which indicates that their functioning may require less fuel and fewer metals. Thanks to nanotechnology, synthetic chemistry has progressed to the extent that it is now possible to create nanoscopic molecules of almost any shape and structure that can be used to prepare a broad range of effective chemicals such as pharmaceuticals and industrial polymers. Nanomedicine is another important application of nanotechnology. Nanofluid is one of the most fundamental and significant units of nanotechnology. During the period of last forty years, nanofluids have piqued the attention of a great number of scientists due to their practical applications and enhanced physical characteristics over conventional fluids.

In this modern era, the class of nanofluids is considered an advanced category of heat transporting fluids because of the potential features and augmented thermal performances of nanofluids. A nanofluid is a combination of some pure fluid like water or oil and nanometer (nm) sized particles (typically 1–100 nm) made of carbon nanotubes (CNTs), oxides, or metals [1]. The development of nanofluids leads to giving rise to a slew of new research directions in numerous sub-disciplines of science and engineering. Choi and Eastman’s pioneering study in which they proposed the concept of nanofluids, is credited for these extensive advancements [2]. Nanofluids contain unique attributes that make them appreciably productive for multiple purposes, for instance, oil purification, production of fiber, accurate drug delivery, manufacturing of plastic and rubber sheets, replacement and repairing of tissues, lubrication, and wear prevention. Apart from this, replacing conventional fluids with nanofluids is one of the best operational strategies for efficient thermal management of various mechanical and industrial systems such as generators, transformers, nuclear reactors, freezers and refrigerators, lawnmowers, electronic equipment, microprocessors, heat exchangers, automobile engines, energy storage instruments, chillers, drilling machines, hydrodynamic pumps, and many others [3]. A complete understanding of nanofluids’ rheological attributes is necessary to determine their viability for heat transport challenges. In the last twenty years, plenty of experimental and computational investigations have been conducted to evaluate different aspects of nanofluids, for example, methods of preparation, enhancement in particular features, uniform and smooth dispersion of nanoparticles, and improvement in the thermal behavior.

Ali et al. [4] experimentally anticipated the enhancement in thermal efficiency of car radiators. They conducted this study for four different volumetric proportions of ZnO nanoparticles in water (0.01%, 0.08%, 0.2%, 0.3%) and witnessed maximum augmentation of heat transfer (i.e., 46%) against 0.2% volume proportion. Saffarian et al. [5] examined various geometries of homogeneous lengths to analyze the influence of a conduit’s shape on the heat transporting tendency of nanofluids. More precisely, they investigated the flow phenomenon through wavy, U-shaped, and spiral conduits. According to their findings, a wavy pipe shaped conduit is the most appropriate geometry among the ones considered, as it increased the heat transportation capacity of base fluid by 78%. Uddin et al. [6] theoretically inspected the two-dimensional slip flow of a nanofluid in a Darcian porous material generated due to the shrinking and stretching motion of a sheet. They utilized a numerical method to solve an intricate nonlinear system of partial differential equations. This study reveals that the heat transfer rate is greater for the case of a shrinking sheet as compared to that of a stretching sheet. The mixed convective flow of ethylene and water based γ-nanofluids over an extended upward surface was studied by Khan et al. [7]. The experimental expressions of viscosity and thermal conductance were used and consequences of radiation and entropy production were also evaluated. It was reported that a substantial advancement in the heat transportation rate appears because of radiation and unsteady parameter. Mahanthesh et al. [8] inspected the impacts of heat injection on viscous nanofluid, which exhibits its motion near a radially stretched disk. Thermophoresis and Brownian motion mechanisms were also incorporated in the model. It was claimed that the influence of thermophoresis on heat transport coefficient is dominant than that of Brownian motion. Sun et al. [9] explored that how the thermal conductivity of iron oxides based water nanofluid, which indicates circular motion inside a tube is altered by flux density, gradient, and different magnetic field orientations. They came to the conclusion that a vertically applied magnetic field causes the formation of chain-like structures, which minimizes thermal resistance and escalates convectional heat transport. Khan et al. [10] carried out a numerical analysis to probe the significance of magnetic dipole and activation energy for nanofluid’s cross-flow and heat transportation in a cylinder. They also discussed that the thermophoretic parameter intensifies the temperature function. Some latest investigations on improved features and better thermal performance of nanofluids are enclosed in [11,12,13,14,15,16,17,18,19,20].

In this century, fractional calculus is a rapidly evolving field because of its widespread applications in a range of real-world scenarios. Several scholars recently claimed that fractional operators provide much more accurate results and have a greater impact on analysis than standard derivatives. With the help of various experimental and theoretical studies, it has been reported that the adaptation of an appropriate fractional parameter results in almost complete consistency between theoretic and experimental findings. In the area of fluid mechanics, certain rheological properties of fluids can only be analyzed using fractional operators. During the duration of the last fifteen years, fractional operators have been effectively utilized for adequate modeling of a wide range of practical operations and processes. The application fields of fractional operators include biophysics, signals processing, epidemic simulations, fluid mechanics, moving wave solutions, rheology, biochemical models, viscoelasticity, economics, and so forth. Viewing the crucial implications of fractional operators, researchers/investigators realized imperative demands to develop some new fractional operators, which guarantee the specificity of the modeling process, possess various novel features, and able to appropriately describe complex real-life phenomena. In this regard, all the proposed fractional operators so far have a unique kernel. For example, Riemann-Liouville and Caputo fractional operators are established in terms of the power-law kernel, which later proved to be a singular one [21]. Caputo and Fabrizio dealt with this singularity problem by devising a new fractional operator made up of a kernel in the form of an exponential decay law [22]. The Atangana-Baleanu fractional operator is the latest introduction to the family of fractional operators, which applies the concept of generalized Mittag-Leffler law to effectively overcome the deficiencies of preceding operators [23]. Since fractional operators are so efficient in simulation and modeling, some researchers are interested in converting problems from regular to fractional environments. Goufo et al. [24] used the Atangana-Baleanu fractional operator to analyze neuronal activities with the support of a three-dimensional Hindmarsh-Rose neuron model. Faraz et al. [25] operated different fractional operators to transform some existing models in order to perform an inclusive dynamical study of COVID-19. They also explicated the efficacy of several precautionary measures such as lockdown and quarantine in preventing the infection’s spread. Tarasov [26] discussed the ongoing development in modern mathematical economics due to fractional operators. Skovranek [27] investigated and explained the effects of unemployment on the inflation rate by developing a mathematical model such that the power function was replaced with the Mittag-Leffler function for the transformation of the problem to a fractional framework. Riaz et al. [28] elucidated how modern fractional operators significantly contribute to describe simultaneously occurring heat flow and transportation phenomena near a vertical wall. Several new analyses outlining the usefulness of fractional operators for flow and heat transfer challenges are documented in [29,30,31,32,33,34,35,36,37].

An extensive literature review discloses that most of the investigations are conducted for nanoparticles of spherical shapes, which not only unable to completely evaluate all the necessary attributes of nanofluids but also limited in terms of practical significance and implications. Keeping a view of these disadvantages, the current study aims to explore the impacts of multiple shapes of nanoparticles. More precisely, brick, cylinder, blade, and platelet shapes of nanoparticles are considered for this work. The literature also communicates that majority of researchers are interested in copper, CNTs, alumina, and titania nanoparticles. However, there exist many other nanoparticles that possess the ability to enrich the thermal characteristics of conventional fluids effectively. Thus, in this work, $MoS_{2}$ nanoparticles are chosen due to their momentous and imperative applications in various fields, particularly in 2D electronic instruments like field-effect transistors and semiconductors. $MoS_{2}$ has a layered structure like graphene with sufficiently large band gaps. Moreover, the improved thermophysical features of $MoS_{2}$ based nanofluids such as augmented specific heat capacitance and thermal conductance, low friction, enhanced lubrication tendency, and robustness make them vitally effective for diverse mechanical applications [38,39]. Realizing these valuable advantages, the current fractional analysis aims to investigate $MoS_{2}$ based sodium alginate nanofluid in a channel frame. Non-integer order PDEs are presented in the form of Caputo-Fabrizio and Atangana-Baleanu fractional operators to describe transportation and heat transfer phenomena. These fractional models are separately solved by utilizing the Laplace transform and a detailed comparative study is conducted to anticipate which operator is more effective for the current problem. Subject to magnetic and radiation effects, a dynamical analysis of considered nanofluid is performed for three different flow situations in a permeable medium. Various parameters and quantities of physical interest are studied through tabulated data and several graphical illustrations of flow patterns and the temperature field.

## 2. Statement and Mathematical Modeling

**Figure 1:**
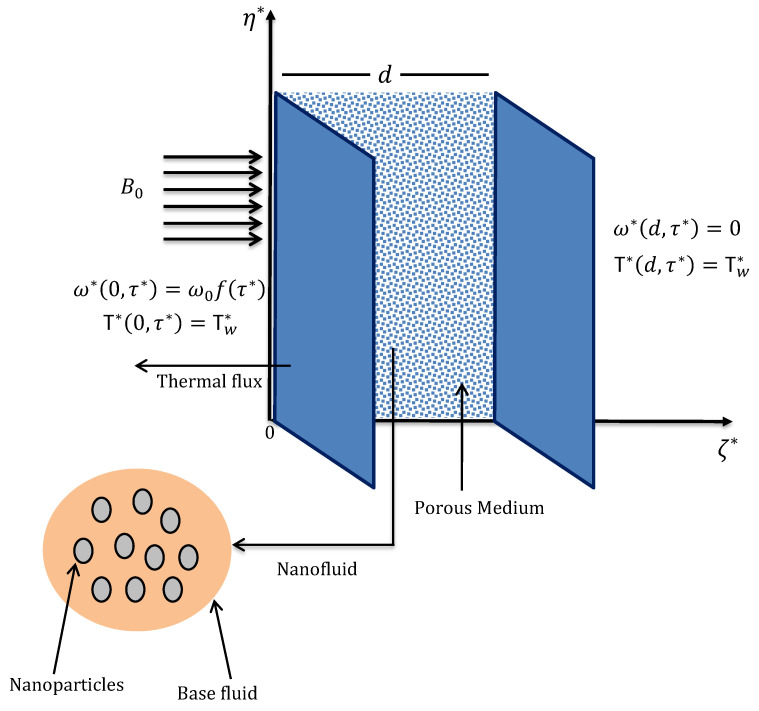
Geometrical model of the considered problem.

Consider magnetohydrodynamic (MHD) convectional flow of sodium alginate based nanofluid between two parallel upward walls (vertical channel) saturated in a permeable material. The left wall is located at the origin and the right wall is situated at a distance *d* as portrayed in Figure 1. A magnetic field and radiative flux are acting on the channel along the horizontal axis (labeled as $\zeta^{*}$) whereas, these effects are assumed insignificant in the vertical direction (labeled as $\eta^{*}$). Depending on the movement of the left wall, the transport phenomenon is observed for three different physical situations. The base fluid sodium alginate contains $MoS_{2}$ nanoparticles of multiple shapes and there exists a state of thermal equilibrium between them. Due to the low Reynolds number, the induced magnetic field is sufficiently small such that it can be neglected. Moreover, polarization effects indicate no contribution to flow dynamics therefore, the electrical field is not yielded externally. Since the flow is along the $\eta^{*}-$axis and transient effects are incorporated in the model, velocity and temperature functions depend on time $\tau^{*}$ and space coordinate $\zeta^{*}$.

The usual Boussinesq approximation [40] is applied to develop the flow equation while the Rosseland approximation [41] is used to deal with radiative heat flux during modeling of the energy equation. These equations are provided as [42,43]

$$\begin{array}{c} {\overset{ˇ}{\rho }}_{nf}\frac{\partial \omega^{*}\left(\zeta^{*},\tau^{*}\right)}{\partial \tau^{*}}=\left(1+\frac{1}{\beta }\right){\overset{ˇ}{\mu }}_{nf}\frac{\partial^{2}\omega^{*}\left(\zeta^{*},\tau^{*}\right)}{\partial \zeta^{*2}}+g{\left(\overset{ˇ}{\rho }{\overset{ˇ}{\beta }}_{1}\right)}_{nf}\left({⊤}^{*}\left(\zeta^{*},\tau^{*}\right)-{⊤}_{d}^{*}\right) \end{array}$$

(1) $$\begin{array}{c} -{\overset{ˇ}{\sigma }}_{nf}B_{0}^{2}\omega^{*}\left(\zeta^{*},\tau^{*}\right)-\left(1+\frac{1}{\beta }\right)\frac{{\overset{ˇ}{\mu }}_{nf}\phi_{p}}{K_{p}}\omega^{*}\left(\zeta^{*},\tau^{*}\right),\;\;\; \end{array}$$

(2) $$\begin{array}{c} {\left(\overset{ˇ}{\rho }{\overset{ˇ}{C}}_{p}\right)}_{nf}\frac{\partial {⊤}^{*}\left(\zeta^{*},\tau^{*}\right)}{\partial \tau^{*}}={\overset{ˇ}{k}}_{nf}\frac{\partial^{2}{⊤}^{*}\left(\zeta^{*},\tau^{*}\right)}{\partial \zeta^{*2}}-\frac{\partial q_{r}}{\partial \zeta^{*}},\;\;\; \end{array}$$

(3) $$\begin{array}{c} \left[q_{r}=-\frac{4\sigma_{1}}{3k_{1}}\frac{\partial {⊤}^{*4}}{\partial \zeta^{*}},\;{⊤}^{*4}\approx \left(4{⊤}_{d}^{{}^{*}3}\right){⊤}^{*}-3{⊤}_{d}^{{}^{*}4}\right].\;\;\; \end{array}$$

Here $\omega^{*}\left(\zeta^{*},\tau^{*}\right)$ denotes the velocity function and ${⊤}^{*}\left(\zeta^{*},\tau^{*}\right)$ represents the temperature of nanofluid. β is known as Casson parameter, $\sigma_{1}$ is Stefan-Boltzmann constant, $B_{0}$ accounts for the strength of magnetic influence, ${⊤}_{d}^{*}$ is initial temperature, *g* characterizes gravity effects, permeability and porosity of the material are symbolized with $K_{p}$ and $\phi_{p}$ respectively, and mean absorption is denoted by $k_{1}$. The employed conditions are mathematically expressed as

(4) $$\begin{array}{c} \omega^{*}\left(\zeta^{*},0\right)=0,\;{⊤}^{*}\left(\zeta^{*},0\right)={⊤}_{d}^{*},\;\frac{\partial \omega^{*}\left(\zeta^{*},0\right)}{\partial \tau^{*}}=0, \end{array}$$

(5) $$\begin{array}{c} \omega^{*}\left(0,\tau^{*}\right)=\omega_{0}f\left(\tau^{*}\right),\;{⊤}^{*}\left(0,\tau^{*}\right)={⊤}_{w}^{*}, \end{array}$$

(6) $$\begin{array}{c} \omega^{*}\left(d,\tau^{*}\right)=0,\;{⊤}^{*}\left(d,\tau^{*}\right)={⊤}_{w}^{*}. \end{array}$$

There exist some thermo-physical quantities in modeled Equations (1)–(3), which dependent on particular features of carrier fluid sodium alginate and $MoS_{2}$ nanoparticles. These quantities are important in determining the overall thermal efficiency and flow pattern of the resulted nanofluid. For precise anticipation of these quantities, particular mathematical expressions are proposed in the literature. Viewing the usefulness of the Hamilton and Crosser model [44], it is employed in this work to compute viscosity (${\overset{ˇ}{\mu }}_{nf}$) and thermal conductivity (${\overset{ˇ}{k}}_{nf}$) of nanofluid in the following manner

(7) $$\begin{array}{c} {\overset{ˇ}{\mu }}_{nf}={\overset{ˇ}{\mu }}_{sa}\left(1+\overset{ˇ}{a}\phi^{2}+\overset{ˇ}{b}\phi \right), \end{array}$$

(8) $$\begin{array}{c} {\overset{ˇ}{k}}_{nf}={\overset{ˇ}{k}}_{sa}\left\{\frac{\left(j-1\right){\overset{ˇ}{k}}_{sa}-\phi \left(j-1\right)\left({\overset{ˇ}{k}}_{sa}-{\overset{ˇ}{k}}_{ms}\right)+{\overset{ˇ}{k}}_{ms}}{\left(j-1\right){\overset{ˇ}{k}}_{sa}+\phi \left({\overset{ˇ}{k}}_{sa}-{\overset{ˇ}{k}}_{ms}\right)+{\overset{ˇ}{k}}_{ms}}\right\}. \end{array}$$

Throughout this manuscript, the subscripts nf, sa, and ms are utilized to indicate characteristics of nanofluid, sodium alginate, and molybdenum-disulfide nanoparticles respectively. In the above equations, ϕ is used to characterize the volume proportion of nanoparticles. The shape constants are denoted with $\overset{ˇ}{a}$ and $\overset{ˇ}{b}$ in Equation (7). On the other end, *j* is the shape factor in Equation (8), and it is represented as j=3/ψ, where ψ is the quotient of surface areas of the sphere and real nanoparticles. Table 1 conveys the operational values of $\overset{ˇ}{a}$, $\overset{ˇ}{b}$, and ψ.

The relations for other properties of nanofluids such as density (${\overset{ˇ}{\rho }}_{nf}$), electrical conductance (${\overset{ˇ}{\sigma }}_{nf}$), heat capacity (${\left(\overset{ˇ}{\rho }{\overset{ˇ}{C}}_{p}\right)}_{nf}$), and thermal expansion (${\left(\overset{ˇ}{\rho }{\overset{ˇ}{\beta }}_{1}\right)}_{nf}$) are provided as [45]

$$\begin{array}{c} {\overset{ˇ}{\rho }}_{nf}=\phi {\overset{ˇ}{\rho }}_{ms}+\left(1-\phi \right){\overset{ˇ}{\rho }}_{sa}, \end{array}$$

$$\begin{array}{c} {\overset{ˇ}{\sigma }}_{nf}={\overset{ˇ}{\sigma }}_{sa}+\frac{3\phi {\overset{ˇ}{\sigma }}_{sa}\left({\overset{ˇ}{\sigma }}_{ms}-{\overset{ˇ}{\sigma }}_{sa}\right)}{{\overset{ˇ}{\sigma }}_{ms}+2{\overset{ˇ}{\sigma }}_{sa}+\phi \left({\overset{ˇ}{\sigma }}_{sa}-{\overset{ˇ}{\sigma }}_{ms}\right)}, \end{array}$$

$$\begin{array}{c} {\left(\overset{ˇ}{\rho }\overset{ˇ}{C_{p}}\right)}_{nf}=\phi {\left(\overset{ˇ}{C_{p}}\overset{ˇ}{\rho }\right)}_{ms}+\left(1-\phi \right){\left(\overset{ˇ}{C_{p}}\overset{ˇ}{\rho }\right)}_{sa}, \end{array}$$

$$\begin{array}{c} {\left(\overset{ˇ}{\rho }\overset{ˇ}{\beta_{1}}\right)}_{nf}=\phi {\left(\overset{ˇ}{\beta_{1}}\overset{ˇ}{\rho }\right)}_{ms}+\left(1-\phi \right){\left(\overset{ˇ}{\beta_{1}}\overset{ˇ}{\rho }\right)}_{sa}. \end{array}$$

Table 2 communicates the operational values for physical and thermal quantities of NaAlg and $MoS_{2}$.

Equations (1)–(3) are treated with the following new set of unit-less quantities to acquire a dimensionless version of the model

(9) $$\begin{array}{c} \zeta =\frac{1}{d}\zeta^{*},\;\omega =\frac{d}{\nu_{sa}}\omega^{*},\;\vartheta =\frac{{⊤}^{*}-{⊤}_{d}^{*}}{{⊤}_{w}^{*}-{⊤}_{d}^{*}},\;\tau =\frac{\nu_{sa}}{d^{2}}\tau^{*}. \end{array}$$

The governing equations and associated conditions are presented in a unit-free form as

(10) $$\begin{array}{c} {\overset{ˇ}{n}}_{4}\frac{\partial \omega (\zeta ,\tau )}{\partial \tau }={\overset{ˇ}{n}}_{8}\frac{\partial^{2}\omega \left(\zeta ,\tau \right)}{\partial \zeta^{2}}+G_{r0}\vartheta \left(\zeta ,\tau \right)-M_{0}\omega \left(\zeta ,\tau \right)-\frac{1}{K_{0}}\omega \left(\zeta ,\tau \right), \end{array}$$

(11) $$\begin{array}{c} \frac{\partial \vartheta (\zeta ,\tau )}{\partial \tau }={\overset{ˇ}{n}}_{3}\frac{\partial^{2}\vartheta \left(\zeta ,\tau \right)}{\partial \zeta^{2}},\;\;\;\;\; \end{array}$$

(12) $$\begin{array}{c} \omega \left(\zeta ,0\right)=0,\;\vartheta \left(\zeta ,0\right)=0,\;\frac{\partial \omega (\zeta ,0)}{\partial \tau }=0,\;\;\; \end{array}$$

(13) $$\begin{array}{c} \omega (0,\tau )=f(\tau ),\;\vartheta (0,\tau )=1,\;\;\;\; \end{array}$$

(14) $$\begin{array}{c} \omega (1,\tau )=0,\;\vartheta (1,\tau )=1,\;\;\;\; \end{array}$$

where

(15) $$\begin{array}{c} {\overset{ˇ}{n}}_{1}=\frac{\left(j-1\right){\overset{ˇ}{k}}_{sa}-\phi \left(j-1\right)\left({\overset{ˇ}{k}}_{sa}-{\overset{ˇ}{k}}_{ms}\right)+{\overset{ˇ}{k}}_{ms}}{\left(j-1\right){\overset{ˇ}{k}}_{sa}+\phi \left({\overset{ˇ}{k}}_{sa}-{\overset{ˇ}{k}}_{ms}\right)+{\overset{ˇ}{k}}_{ms}},\;{\overset{ˇ}{n}}_{2}=\left(1-\phi \right)+\frac{\phi {\left(\overset{ˇ}{\rho }\overset{ˇ}{C_{p}}\right)}_{ms}}{{\left(\overset{ˇ}{\rho }\overset{ˇ}{C_{p}}\right)}_{sa}},\;\\ {\overset{ˇ}{n}}_{3}=\frac{{\overset{ˇ}{n}}_{1}}{{\overset{ˇ}{n}}_{2}}\frac{1}{P_{r0}},\;{\overset{ˇ}{n}}_{4}=\left(1-\phi \right)+\frac{\phi {\overset{ˇ}{\rho }}_{ms}}{{\overset{ˇ}{\rho }}_{sa}},\;{\overset{ˇ}{n}}_{5}=\left(1+\overset{ˇ}{a}\phi^{2}+\overset{ˇ}{b}\phi \right),\;\;\\ {\overset{ˇ}{n}}_{6}=\left(1-\phi \right)+\frac{\phi {\left(\overset{ˇ}{\rho }\overset{ˇ}{\beta_{1}}\right)}_{ms}}{{\left(\overset{ˇ}{\rho }\overset{ˇ}{\beta_{1}}\right)}_{sa}},\;{\overset{ˇ}{n}}_{7}=1+\frac{3\phi ({\overset{ˇ}{\sigma }}_{2}-1)}{\left({\overset{ˇ}{\sigma }}_{2}+2\right)-\left({\overset{ˇ}{\sigma }}_{2}-1\right)\phi },\;\;\\ {\overset{ˇ}{n}}_{8}={\overset{ˇ}{n}}_{5}\left(1+\frac{1}{{\overset{ˇ}{\beta }}_{1}}\right),\;P_{r}={\left(\frac{\overset{ˇ}{\mu }{\overset{ˇ}{C}}_{p}}{\overset{ˇ}{k}}\right)}_{sa},\;P_{r0}=\frac{{\overset{ˇ}{n}}_{1}P_{r}}{{\overset{ˇ}{n}}_{1}+Nr},\;\;\;\\ Nr=\frac{16\sigma_{2}{⊤}_{\infty }^{3}}{3k_{2}{\overset{ˇ}{k}}_{sa}},\;M=\frac{\overset{ˇ}{\sigma }B_{0}d^{2}}{\overset{ˇ}{\rho }\overset{ˇ}{\nu }},\;M_{0}={\overset{ˇ}{n}}_{7}M,\;{\overset{ˇ}{\sigma }}_{2}=\frac{{\overset{ˇ}{\sigma }}_{ms}}{{\overset{ˇ}{\sigma }}_{sa}},\;\;\\ \;\frac{1}{K}=\frac{\phi_{p}d^{2}}{K_{p}},\;G_{r0}={\overset{ˇ}{n}}_{6}Gr,\;G_{r1}=\frac{G_{r0}}{{\overset{ˇ}{n}}_{8}},\;\;\;\\ \frac{1}{K_{0}}={\overset{ˇ}{n}}_{5}\left(1+\frac{1}{{\overset{ˇ}{\beta }}_{1}}\right)\frac{1}{K},\;Gr=\frac{g{\overset{ˇ}{\beta }}_{1}\left({⊤}_{w}-{⊤}_{d}\right)d^{3}}{{\overset{ˇ}{\nu }}^{2}},\;\; \end{array}$$

where *M*, Nr, and *K*, are the parameters of magnetic, radiation, and porosity respectively. $P_{r}$ and Gr are called Prandtl and Grashof number respectively, and ${\overset{ˇ}{n}}_{i}\left(i=1,\ldots ,8\right)$ are some constants.

## 3. Fractional Models and Analytic Solutions

This section aims to formulate two different fractional models in terms of Atangana-Baleanu and Caputo-Fabrizio fractional operators. Before developing these models, it is necessary to define these operators and their Laplace transform as the solution finding methodology involves the employment of the Laplace transformation (LT) technique. The fractional operators named Atangana-Baleanu and Caputo-Fabrizio for an arbitrary function V(ζ,τ) and non-integer order Γ∈(0,1) are respectively given as [22,23]

(16) $$\begin{array}{c} {}^{AB}D_{\tau }^{\Gamma }\left\{V\left(\zeta ,\tau \right)\right\}=\frac{1}{1-\Gamma }\int_{0}^{\tau }\frac{\partial V(\zeta ,s)}{\partial s}E_{\Gamma }\left(-\Gamma \frac{{\left(\tau -s\right)}^{\Gamma }}{1-\Gamma }\right)ds, \end{array}$$

(17) $$\begin{array}{c} {}^{CF}D_{\tau }^{\Gamma }\left\{V\left(\zeta ,\tau \right)\right\}=\frac{1}{1-\Gamma }\int_{0}^{\tau }\frac{\partial V(\zeta ,s)}{\partial s}\exp\left(-\Gamma \frac{\tau -s}{1-\Gamma }\right)ds, \end{array}$$

where $E_{\Gamma }$ is the generalized Mittag-Leffler function. The LT of Equations (16) and (17) is computed as

(18) $$\begin{array}{c} {}^{AB}L\left\{D_{\tau }^{\Gamma }V\left(\zeta ,\tau \right)\right\}\left(q\right)=\frac{q^{\Gamma }L\left\{V(\zeta ,\tau )\right\}-q^{\left(1-\Gamma \right)}V\left(\zeta ,0\right)}{\Gamma +\left(1-\Gamma \right)q^{\Gamma }}, \end{array}$$

(19) $$\begin{array}{c} {}^{CF}L\left\{D_{\tau }^{\Gamma }V\left(\zeta ,\tau \right)\right\}\left(q\right)=\frac{qL\left\{V(\zeta ,\tau )\right\}-V\left(\zeta ,0\right)}{\Gamma +(1-\Gamma )q}.\; \end{array}$$

### 3.1. Temperature Field

#### 3.1.1. Atangana-Baleanu Fractional Operator

The non-integer order energy equation is obtained by incorporating the Atangana-Baleanu operator at the place of ordinary time derivative in Equation (11) as

(20) $$\begin{array}{c} {}^{AB}D_{\tau }^{\Gamma }\left\{\vartheta (\zeta ,\tau )\right\}={\overset{ˇ}{n}}_{3}\frac{\partial^{2}\vartheta \left(\zeta ,\tau \right)}{\partial \zeta^{2}}. \end{array}$$

On implementation of the LT, Equation (20) adopts the following form

(21) $$\begin{array}{c} \frac{d^{2}\bar{\vartheta }\left(\zeta ,q\right)}{d\zeta^{2}}-\frac{q^{\Gamma }\bar{\vartheta }\left(\zeta ,q\right)}{{\overset{ˇ}{n}}_{3}\left\{q^{\Gamma }\left(1-\Gamma \right)+\Gamma \right\}}=0, \end{array}$$

Simplifying Equation (21) and applying the LT on corresponding boundary conditions (13) and (14) yield the following system

(22) $$\begin{array}{c} \frac{d^{2}\bar{\vartheta }\left(\zeta ,q\right)}{d\zeta^{2}}-\frac{q^{\Gamma }{\overset{ˇ}{h}}_{0}\bar{\vartheta }\left(\zeta ,q\right)}{{\overset{ˇ}{n}}_{3}\left\{q^{\Gamma }+{\overset{ˇ}{f}}_{0}\right\}}=0, \end{array}$$

(23) $$\begin{array}{c} \bar{\vartheta }\left(0,q\right)=\frac{1}{q},\;\bar{\vartheta }\left(1,q\right)=\frac{1}{q},\; \end{array}$$

where

$$\begin{array}{c} {\overset{ˇ}{h}}_{0}=\frac{1}{1-\Gamma }\;\mathrm{and}\;{\overset{ˇ}{f}}_{0}=\frac{\Gamma }{1-\Gamma }. \end{array}$$

On solving the system presented in Equations (22) and (23), the solution is calculated as

(24) $$\begin{array}{c} \bar{\vartheta }\left(\zeta ,q\right)=\frac{1-\exp\left(-\sqrt{R}\right)}{2\left(\sinh(\sqrt{R})\right)q}\exp\left(\sqrt{R}\zeta \right)+\frac{\exp\left(\sqrt{R}\right)-1}{2\left(\sinh(\sqrt{R})\right)q}\exp\left(-\sqrt{R}\zeta \right), \end{array}$$

where

$$\begin{array}{c} \;R=\frac{{\overset{ˇ}{h}}_{0}q^{\Gamma }}{{\overset{ˇ}{n}}_{3}\left\{q^{\Gamma }+{\overset{ˇ}{f}}_{0}\right\}}. \end{array}$$

The heat flow rate at the interface is quantified in terms of Nusselt number as

(25) $$\begin{array}{c} Nu=-{\overset{ˇ}{n}}_{1}\frac{\partial \vartheta (\zeta ,\tau )}{\partial \zeta }{|}_{\zeta =0}. \end{array}$$

Computing the gradient of temperature function by utilizing Equation (24) and substituting it in Equation (25) return the final expression of Nusselt number as

(26) $$\begin{array}{c} Nu=-{\overset{ˇ}{n}}_{1}\sqrt{R}\left[\left(\frac{1-\exp\left(-\sqrt{R}\right)}{2\left(\sinh(\sqrt{R})\right)q}\right)-\left(\frac{\exp\left(\sqrt{R}\right)-1}{2\left(\sinh(\sqrt{R})\right)q}\right)\right]. \end{array}$$

#### 3.1.2. Caputo-Fabrizio Fractional Operator

In this section, a mathematical relation for energy function is computed to develop the temperature field in terms of the Caputo-Fabrizio operator. For this purpose, the ordinary time derivative in Equation (11) is substituted with the Caputo-Fabrizio time derivative as

(27) $$\begin{array}{c} {}^{CF}D_{\tau }^{\Gamma }\left\{\vartheta (\zeta ,\tau )\right\}={\overset{ˇ}{n}}_{3}\frac{\partial^{2}\vartheta \left(\zeta ,\tau \right)}{\partial \zeta^{2}}. \end{array}$$

Application of the LT on Equation (27) gives

(28) $$\begin{array}{c} \frac{d^{2}\bar{\vartheta }\left(\zeta ,q\right)}{d\zeta^{2}}-\frac{q\bar{\vartheta }\left(\zeta ,q\right)}{{\overset{ˇ}{n}}_{3}\left\{q\left(1-\Gamma \right)+\Gamma \right\}}=0, \end{array}$$

The above equation is simplified and presented in the following way

(29) $$\begin{array}{c} \frac{d^{2}\bar{\vartheta }\left(\zeta ,q\right)}{d\zeta^{2}}-\frac{q{\overset{ˇ}{h}}_{0}\bar{\vartheta }\left(\zeta ,q\right)}{{\overset{ˇ}{n}}_{3}\left\{q+{\overset{ˇ}{f}}_{0}\right\}}=0, \end{array}$$

The solution of Equation (29) is established in the presence of boundary conditions given in Equation (23) as

(30) $$\begin{array}{c} \bar{\vartheta }\left(\zeta ,q\right)=\frac{1-\exp\left(-\sqrt{S}\right)}{2\left(\sinh(\sqrt{S})\right)q}\exp\left(\sqrt{S}\zeta \right)+\frac{\exp\left(\sqrt{S}\right)-1}{2\left(\sinh(\sqrt{S})\right)q}\exp\left(-\sqrt{S}\zeta \right), \end{array}$$

where

$$\begin{array}{c} S=\frac{{\overset{ˇ}{h}}_{0}q}{{\overset{ˇ}{n}}_{3}\left\{q+{\overset{ˇ}{f}}_{0}\right\}}. \end{array}$$

In this case, Nusselt number is obtained as

(31) $$\begin{array}{c} Nu=-{\overset{ˇ}{n}}_{1}\sqrt{S}\left[\left(\frac{1-\exp\left(-\sqrt{S}\right)}{2\left(\sinh(\sqrt{S})\right)q}\right)-\left(\frac{\exp\left(\sqrt{S}\right)-1}{2\left(\sinh(\sqrt{S})\right)q}\right)\right]. \end{array}$$

### 3.2. Velocity Field

#### 3.2.1. Atangana-Baleanu Fractional Operator

The non-integer order unit-free form of flow Equation (10) is presented as

(32) $$\begin{array}{c} {\overset{ˇ}{n}}_{4}\left[{}^{AB}D_{\tau }^{\Gamma }\left\{\omega (\zeta ,\tau )\right\}\right]={\overset{ˇ}{n}}_{8}\frac{\partial^{2}\omega \left(\zeta ,\tau \right)}{\partial \zeta^{2}}+G_{r0}\vartheta \left(\zeta ,\tau \right)-M_{0}\omega \left(\zeta ,\tau \right)-\frac{1}{K_{0}}\omega \left(\zeta ,\tau \right). \end{array}$$

On employment of the LT, Equation (32) takes the following form

(33) $$\begin{array}{c} {\overset{ˇ}{n}}_{4}\left[\frac{q^{\Gamma }\left\{\bar{\omega }\left(\zeta ,q\right)\right\}}{q^{\Gamma }\left(1-\Gamma \right)+\Gamma }\right]={\overset{ˇ}{n}}_{8}\frac{d^{2}\bar{\omega }\left(\zeta ,q\right)}{d\zeta^{2}}+G_{r0}\bar{\vartheta }\left(\zeta ,q\right)-M_{0}\bar{\omega }\left(\zeta ,q\right)-\frac{1}{K_{0}}\bar{\omega }\left(\zeta ,q\right). \end{array}$$

Equation (33) is modified to obtain a suitable expression for finding the solution. The resulted expression is given as

(34) $$\begin{array}{c} \frac{d^{2}\bar{\omega }\left(\zeta ,q\right)}{d\zeta^{2}}-{\overset{ˇ}{n}}_{9}\bar{\omega }\left(\zeta ,q\right)=-G_{r1}\bar{\vartheta }\left(\zeta ,q\right), \end{array}$$

where

$$\begin{array}{c} {\overset{ˇ}{n}}_{9}=\frac{{\overset{ˇ}{n}}_{4}}{{\overset{ˇ}{n}}_{8}}\frac{q^{\Gamma }{\overset{ˇ}{c}}_{1}+{\overset{ˇ}{c}}_{2}}{\left(q^{\Gamma }+{\overset{ˇ}{f}}_{0}\right){\overset{ˇ}{h}}_{0}},\;{\overset{ˇ}{c}}_{1}=K_{0}\left({\overset{ˇ}{h}}_{0}+M_{0}\right)+1,\;{\overset{ˇ}{c}}_{2}=M_{0}K_{0}{\overset{ˇ}{h}}_{0}+{\overset{ˇ}{f}}_{0}. \end{array}$$

Substitution of Equation (24) in Equation (34) returns

(35) $$\begin{array}{cc} \frac{d^{2}\bar{\omega }\left(\zeta ,q\right)}{d\zeta^{2}}-{\overset{ˇ}{n}}_{9}\bar{\omega }\left(\zeta ,q\right) & =-G_{r1}\left\{\frac{1-\exp\left(-\sqrt{R}\right)}{2\left(\sinh(\sqrt{R})\right)q}\exp\left(\sqrt{R}\zeta \right)\right\}\\ \; & -G_{r1}\left\{\frac{\exp\left(\sqrt{R}\right)-1}{2\left(\sinh(\sqrt{R})\right)q}\exp\left(-\sqrt{R}\zeta \right)\right\}. \end{array}$$

The Laplace transformed version of flow boundary conditions given in Equations (13) and (14) is

(36) $$\begin{array}{c} \bar{\omega }\left(0,q\right)=\bar{f}\left(q\right),\;\bar{\omega }\left(1,q\right)=0. \end{array}$$

Equation (35) is solved with the help of boundary conditions presented in Equation (36) and the solution is provided as

(37) $$\begin{array}{cc} \bar{\omega }\left(\zeta ,q\right) & =\frac{-\left(A_{3}+A_{4}+A_{5}\right)}{2\sinh\left(\sqrt{{\overset{ˇ}{n}}_{9}}\right)}\exp\left(\sqrt{{\overset{ˇ}{n}}_{9}}\zeta \right)+\frac{\left(A_{3}+A_{4}+A_{6}\right)}{2\sinh\left(\sqrt{{\overset{ˇ}{n}}_{9}}\right)}\exp\left(-\sqrt{{\overset{ˇ}{n}}_{9}}\zeta \right)\\ \; & -\left\{\frac{G_{r1}}{R-{\overset{ˇ}{n}}_{9}}\right\}\frac{1-\exp\left(-\sqrt{R}\right)}{2\left(\sinh(\sqrt{R})\right)q}\exp\left(\sqrt{R}\zeta \right)\\ \; & -\left\{\frac{G_{r1}}{R-{\overset{ˇ}{n}}_{9}}\right\}\frac{\exp\left(\sqrt{R}\right)-1}{2\left(\sinh(\sqrt{R})\right)q}\exp\left(-\sqrt{R}\zeta \right). \end{array}$$

Equation (37) is further simplified as

(38) $$\begin{array}{cc} \bar{\omega }\left(\zeta ,q\right) & =\frac{-\left(A_{3}+A_{4}+A_{5}\right)}{2\sinh\left(\sqrt{{\overset{ˇ}{n}}_{9}}\right)}\exp\left(\sqrt{{\overset{ˇ}{n}}_{9}}\zeta \right)\\ & +\frac{\left(A_{3}+A_{4}+A_{5}\right)}{2\sinh\left(\sqrt{{\overset{ˇ}{n}}_{9}}\right)}\exp\left(-\sqrt{{\overset{ˇ}{n}}_{9}}\zeta \right)\\ & -A_{1}\exp\left(\sqrt{R}\zeta \right)-A_{2}\exp\left(-\sqrt{R}\zeta \right), \end{array}$$

where

$$\begin{array}{c} A_{1}=\frac{G_{r1}R_{1}}{R-{\overset{ˇ}{n}}_{9}},\;A_{2}=\frac{G_{r1}R_{2}}{R-{\overset{ˇ}{n}}_{9}},\;A_{3}=A_{1}\exp\left(\sqrt{R}\right),\;A_{4}=A_{2}\exp\left(-\sqrt{R}\right),\\ A_{5}=\left(\bar{f}\left(q\right)-A_{1}-A_{2}\right)\exp\left(-\sqrt{{\overset{ˇ}{n}}_{9}}\right),\;A_{6}=\left(\bar{f}\left(q\right)-A_{1}-A_{2}\right)\exp\left(\sqrt{{\overset{ˇ}{n}}_{9}}\right),\;\\ R_{1}=\frac{1-\exp\left(-\sqrt{R}\right)}{2\left(\sinh(\sqrt{R})\right)q},\;R_{2}=\frac{\exp\left(\sqrt{R}\right)-1}{2\left(\sinh(\sqrt{R})\right)q}.\;\;\; \end{array}$$

Shear stress at the interface is measured in the form of skin friction coefficient as

(39) $$\begin{array}{c} C_{f}={\overset{ˇ}{n}}_{5}\left(\frac{\beta +1}{\beta }\right)\frac{\partial \omega (\zeta ,\tau )}{\partial \zeta }{|}_{\zeta =0}. \end{array}$$

The gradient of velocity function is derived through Equation (38) and substituted in Equation (39) to acquire the final form of skin friction coefficient as

(40) $$\begin{array}{c} C_{f}={\overset{ˇ}{n}}_{5}\left(\frac{\beta +1}{\beta }\right)\left[\sqrt{\overset{ˇ}{n_{9}}}\left\{\frac{-\left(A_{3}+A_{4}+A_{5}\right)}{2\sinh\left(\sqrt{{\overset{ˇ}{n}}_{9}}\right)}-\frac{\left(A_{3}+A_{4}+A_{6}\right)}{2\sinh\left(\sqrt{{\overset{ˇ}{n}}_{9}}\right)}\right\}+\sqrt{R}\left(A_{1}-A_{2}\right)\right]. \end{array}$$

#### 3.2.2. Caputo-Fabrizio Fractional Operator

The fractional form of flow Equation (10) in terms of Caputo-Fabrizio time derivative is

(41) $$\begin{array}{c} {\overset{ˇ}{n}}_{4}\left[{}^{CF}D_{\tau }^{\Gamma }\left\{\omega (\zeta ,\tau )\right\}\right]={\overset{ˇ}{n}}_{8}\frac{\partial^{2}\omega \left(\zeta ,\tau \right)}{\partial \zeta^{2}}+G_{r0}\vartheta \left(\zeta ,\tau \right)-M_{0}\omega \left(\zeta ,\tau \right)-\frac{1}{K_{0}}\omega \left(\zeta ,\tau \right), \end{array}$$

On application of the LT in the light of Equation (19), we obtain

(42) $$\begin{array}{c} {\overset{ˇ}{n}}_{4}\left[\frac{q\{\bar{\omega }\left(\zeta ,q\right)\}}{q(1-\Gamma )+\Gamma }\right]={\overset{ˇ}{n}}_{8}\frac{d^{2}\bar{\omega }\left(\zeta ,q\right)}{d\zeta^{2}}+G_{r0}\bar{\vartheta }\left(\zeta ,q\right)-M_{0}\bar{\omega }\left(\zeta ,q\right)-\frac{1}{K_{0}}\bar{\omega }\left(\zeta ,q\right), \end{array}$$

Further simplification of Equation (42) yields

(43) $$\begin{array}{c} \frac{d^{2}\bar{\omega }\left(\zeta ,q\right)}{d\zeta^{2}}-{\overset{ˇ}{n}}_{9}\bar{\omega }\left(\zeta ,q\right)=-G_{r1}\bar{\vartheta }\left(\zeta ,q\right), \end{array}$$

where

$$\begin{array}{c} {\overset{ˇ}{m}}_{9}=\frac{{\overset{ˇ}{n}}_{4}}{{\overset{ˇ}{n}}_{8}}\frac{q{\overset{ˇ}{c}}_{1}+{\overset{ˇ}{c}}_{2}}{\left(q+{\overset{ˇ}{f}}_{0}\right){\overset{ˇ}{h}}_{0}},\;{\overset{ˇ}{c}}_{1}=K_{0}\left({\overset{ˇ}{h}}_{0}+M_{0}\right)+1,\;{\overset{ˇ}{c}}_{2}=M_{0}K_{0}{\overset{ˇ}{h}}_{0}+{\overset{ˇ}{f}}_{0}. \end{array}$$

Plugging Equation (30) in Equation (43) gives

(44) $$\begin{array}{cc} \frac{d^{2}\bar{\omega }\left(\zeta ,q\right)}{d\zeta^{2}}-{\overset{ˇ}{m}}_{9}\bar{\omega }\left(\zeta ,q\right) & =-G_{r1}\left\{\frac{1-\exp\left(-\sqrt{S}\right)}{2\left(\sinh(\sqrt{S})\right)q}\exp\left(\sqrt{S}\zeta \right)\right\}\\ \; & -G_{r1}\left\{\frac{\exp\left(\sqrt{S}\right)-1}{2\left(\sinh(\sqrt{S})\right)q}\exp\left(-\sqrt{S}\zeta \right)\right\}. \end{array}$$

The solution of Equation (44) with respect to conditions given in Equation (36) is developed as

(45) $$\begin{array}{cc} \bar{\omega }\left(\zeta ,q\right) & =\frac{-\left(B_{3}+B_{4}+B_{5}\right)}{2\sinh\left(\sqrt{{\overset{ˇ}{m}}_{9}}\right)}\exp\left(\sqrt{{\overset{ˇ}{m}}_{9}}\zeta \right)+\frac{\left(B_{3}+B_{4}+B_{6}\right)}{2\sinh\left(\sqrt{{\overset{ˇ}{m}}_{9}}\right)}\exp\left(-\sqrt{{\overset{ˇ}{m}}_{9}}\zeta \right)\\ \; & -\left\{\frac{G_{r1}}{S-{\overset{ˇ}{m}}_{9}}\right\}\frac{1-\exp\left(-\sqrt{S}\right)}{2\left(\sinh(\sqrt{S})\right)q}\exp\left(\sqrt{S}\zeta \right)\\ \; & -\left\{\frac{G_{r1}}{S-{\overset{ˇ}{m}}_{9}}\right\}\frac{\exp\left(\sqrt{S}\right)-1}{2\left(\sinh(\sqrt{S})\right)q}\exp\left(-\sqrt{S}\zeta \right). \end{array}$$

Equation (45) is rearranged as follows

(46) $$\begin{array}{cc} \bar{\omega }\left(\zeta ,q\right) & =\frac{-\left(B_{3}+B_{4}+B_{5}\right)}{2\sinh\left(\sqrt{{\overset{ˇ}{m}}_{9}}\right)}\exp\left(\sqrt{{\overset{ˇ}{m}}_{9}}\zeta \right)\\ \; & +\frac{(B_{3}+B_{4}+B_{5})}{2\sinh\left(\sqrt{{\overset{ˇ}{m}}_{9}}\right)}\exp\left(-\sqrt{{\overset{ˇ}{m}}_{9}}\zeta \right)\\ \; & -B_{1}\exp\left(\sqrt{S}\zeta \right)-B_{2}\exp\left(-\sqrt{S}\zeta \right), \end{array}$$

where

$$\begin{array}{c} B_{1}=\frac{G_{r1}S_{1}}{R-{\overset{ˇ}{m}}_{9}},\;B_{2}=\frac{G_{r1}S_{2}}{R-{\overset{ˇ}{m}}_{9}},\;B_{3}=A_{1}\exp\left(\sqrt{S}\right),\;B_{4}=B_{2}\exp\left(-\sqrt{S}\right),\\ B_{5}=\left(\bar{f}\left(q\right)-B_{1}-B_{2}\right)\exp\left(-\sqrt{{\overset{ˇ}{m}}_{9}}\right),\;B_{6}=\left(\bar{f}\left(q\right)-B_{1}-B_{2}\right)\exp\left(\sqrt{{\overset{ˇ}{m}}_{9}}\right),\;\\ S_{1}=\frac{1-\exp\left(-\sqrt{S}\right)}{2\left(\sinh(\sqrt{S})\right)q},\;S_{2}=\frac{\exp\left(\sqrt{S}\right)-1}{2\left(\sinh(\sqrt{S})\right)q}.\;\;\; \end{array}$$

The skin friction coefficient for this case is determined as

(47) $$\begin{array}{c} C_{f}={\overset{ˇ}{n}}_{5}\left(\frac{\beta +1}{\beta }\right)\left[\sqrt{S}\left(B_{1}-B_{2}\right)\sqrt{{\overset{ˇ}{m}}_{9}}+\left\{\frac{-\left(B_{3}+B_{4}+B_{5}\right)}{2\sinh\left(\sqrt{{\overset{ˇ}{m}}_{9}}\right)}-\frac{\left(B_{3}+B_{4}+B_{6}\right)}{2\sinh\left(\sqrt{{\overset{ˇ}{m}}_{9}}\right)}\right\}\right]. \end{array}$$

The temperature and velocity expressions in Equations (24), (30), (38) and (46) for two different fractional models involve multiple complex functions of Laplace frequency “*q*”. Thus, the present form of these expressions is not optimal for inverting them in real-time coordinate analytically. Generally, these situations occur in the case of fractional models because the application of the Laplace transform on non-integer order equations yields much complicated relations as compared to conventional models. When such complexities appear in engineering problems, the use of numerical Laplace transform is the most powerful strategy for dealing with them. In this work, the solutions are transmuted back to the principal domain by operating the well-known Zakian’s Laplace inversion algorithm [47]. The objectives of certifying the developed solutions and ensuring reliability are achieved by the dint of Stehfest’s [48] and Durbin’s algorithms [49]. The same methodology is followed to obtain the tabulated values of Nusselt number and skin friction coefficient.

## 4. Results and Discussion

In this work, the evaluation of thermal and physical features of sodium alginate due to the addition of $MoS_{2}$ nanoparticles is studied. This purpose is served via fractional modeling of the problem and results are compared for Atangana-Baleanu and Caputo-Fabrizio operators based models. Semi-exact solutions for these two fractional models are computed by jointly employing the Laplace transform and Zakian’s numerical inversion method. Realizing the importance of shape effects, it is considered that nanoparticles have four different shapes (brick, blade, cylinder, and platelet). Moreover, the under observation nanofluid encounters the influence of a porous medium, magnetic field, and thermal radiation. The process of heat transfer and flow of nanofluid are taking place in a micro-channel comprised of parallel vertical walls. The aim of this section is to conduct a detailed analysis regarding thermal performance and flow behavior of considered nanofluid. In this respect, several graphical demonstrations are provided for both fractional models. Additionally, Nusselt number and skin friction are also examined through a comprehensive tabular study.

**Figure 2:**
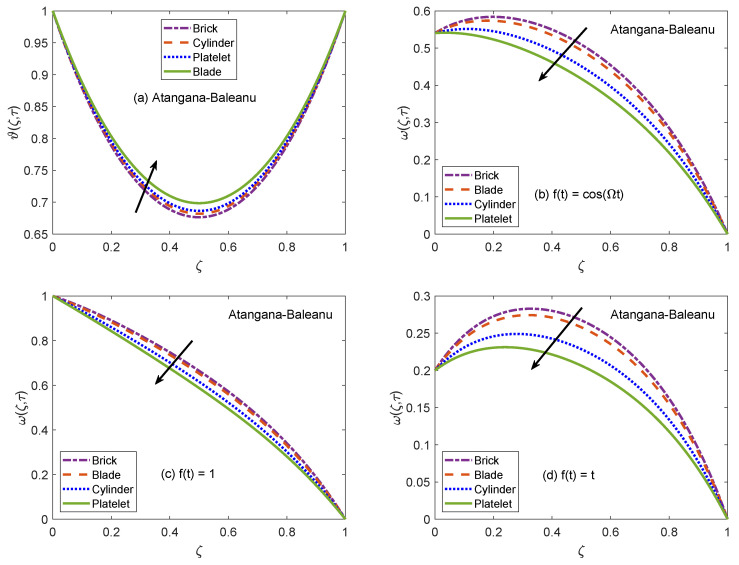
Temperature and velocity patterns of AB model for various shapes of nanoparticles.

**Figure 3:**
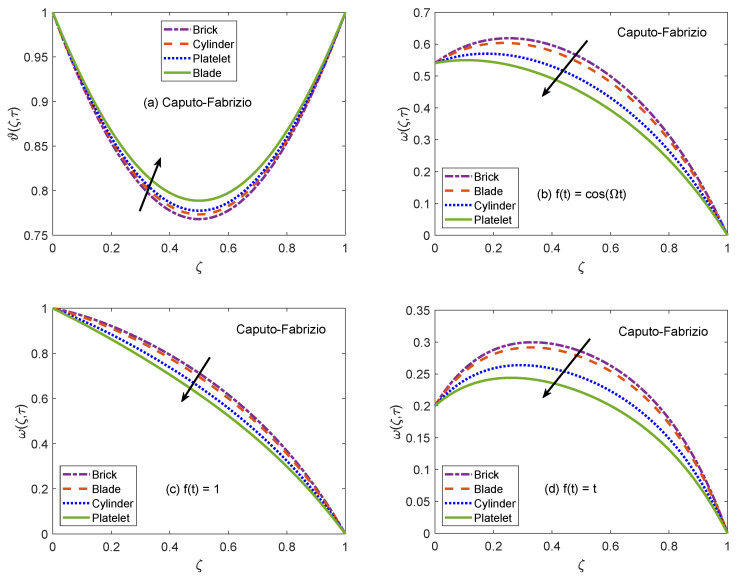
Temperature and velocity patterns of CF model for various shapes of nanoparticles.

**Figure 4:**
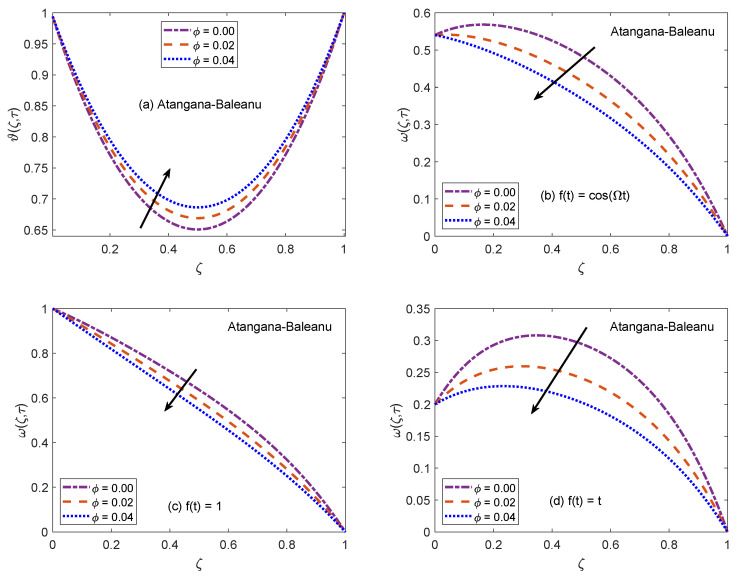
Temperature and velocity patterns of AB model for variation of ϕ.

**Figure 5:**
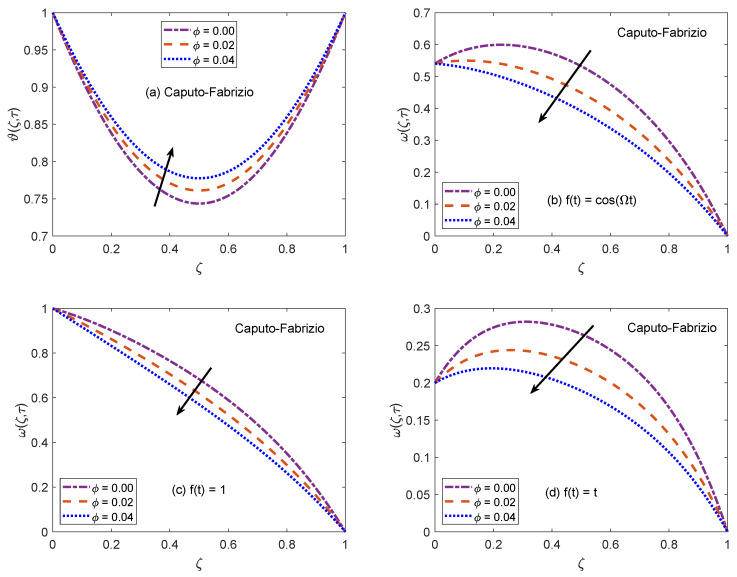
Temperature and velocity patterns of CF model for variation of ϕ.

Figure 2 and Figure 3 communicate the profiles of energy and velocity distributions for AB and CF operators based models. Furthermore, the impacts of shape factor are also studied through these figures. It is observed that nanofluid possesses the minimum energy function for brick shaped nanoparticles and this function attains the highest value when nanoparticles of blade shapes are dispersed. Mainly, this variation is caused by shape factor *j*. In the physical sense, thermal conductivity of nanofluid changes because *j* has a particular value for a specific shape of nanoparticles (as expressed in Equation (8)). Hence, corresponding to change of shapes, the thermal profile of nanofluid indicates a noteworthy variation, which highlights that shape factor plays a significant contribution in the development of temperature field. Furthermore, the CF model yields higher temperature profile as compared to that of AB model. Now, moving towards transport phenomenon, the flow of nanofluid is investigated for three different physical situations. Figure 2b and Figure 3b are for the case when the left wall shows oscillatory motion (f(t)=cos(Ωt)), Figure 2c and Figure 3c are for the constant motion (f(t)=1), and Figure 2d and Figure 3d are for the condition when the wall moves with single acceleration (f(t)=t). It is perceived that the addition of platelet shaped nanoparticles causes a substantial retardation in the flow velocity. Whereas, the swiftest flow of nanofluid is observed for brick shapes nanoparticles. The primary factor behind this behavior is modification of viscosity due to shape constants $\overset{ˇ}{a}$ and $\overset{ˇ}{b}$. Every shape of nanoparticles specifies fixed values of $\overset{ˇ}{a}$ and $\overset{ˇ}{b}$, which lead to defining the viscosity of nanofluid. An interesting fact is witnessed here that the velocity function exhibits the highest profile when the left wall indicates a constant motion. The whole discussion about Figure 2 and Figure 3 can be summarized in the following way that the values of flow and temperature functions are maximum for brick and blade shapes of nanoparticles respectively. The experimental investigation of Timofeeva et al. [50] supports the aforementioned results. They observed that the flow velocity of nanofluids is maximum when the dispersed nanoparticles are of brick shape because the flow resistive capacity of nanofluids is minimum in this case. Furthermore, an investigation on different shapes of $MoS_{2}$ nanoparticles was conducted by Khan [51] and the results of this study show a perfect agreement with the present work. He analyzed that nanofluid with blade and platelet shape $MoS_{2}$ nanoparticles indicate higher thermal profiles as compared to cylinder and brick shape nanoparticles. The total concentration of nanoparticles in base fluid is denoted by ϕ. Figure 4 is developed to analyze the behavior of temperature and flow functions when this concentration is increased to 4% (ϕ=0.04). The respective figure is prepared for AB model and the same analysis is carried out for CF model through Figure 5. It is realized that temperature and flow functions behave in reverse manners. More precisely, the thermal curve is elevated for larger values of ϕ whereas, the flow curve is declined. One of the vital features of nanoparticles is that they enhance the thermal properties of ordinary fluids. Heat capacitance and thermal conductivity are examples of these properties. So, in the current case, when $MoS_{2}$ nanoparticles are added in base fluid sodium alginate, the potential of resulted nanofluid to conduct the heat is much greater as compared to that of pure sodium alginate. Therefore, the thermal curve of nanofluid is higher than the curve of pure fluid (ϕ=0.0). On the other end, these nanoparticles also affect the physical characteristics of conventional fluids such as viscosity and density. In general, the addition of nanoparticles produce more viscous nanofluids. Similarly, it is seen that pure sodium alginate has greater velocity as equated to velocity of $MoS_{2}$ based nanofluid. It is also clear that effects of ϕ variation are significant when the wall moves with single acceleration. Moreover, it is important to discuss here the experimental work of Colla et al. [52]. They performed experimental studies to anticipate and analyze the modifications in the host fluid’s viscosity because of the addition of nanoparticles. They reported a significant rise in thermal conductivity and viscosity of consequent nanofluid due to an enhancement in the loading range of nanoparticles. This conclusion also endorses the opposite behaviors of thermal and flow profiles of the nanofluid considered in the under observation problem. These results highlight the useful applications of nanofluids for thermal management of devices, lubrication, and cooling of advanced industrial operational systems.

**Figure 6:**
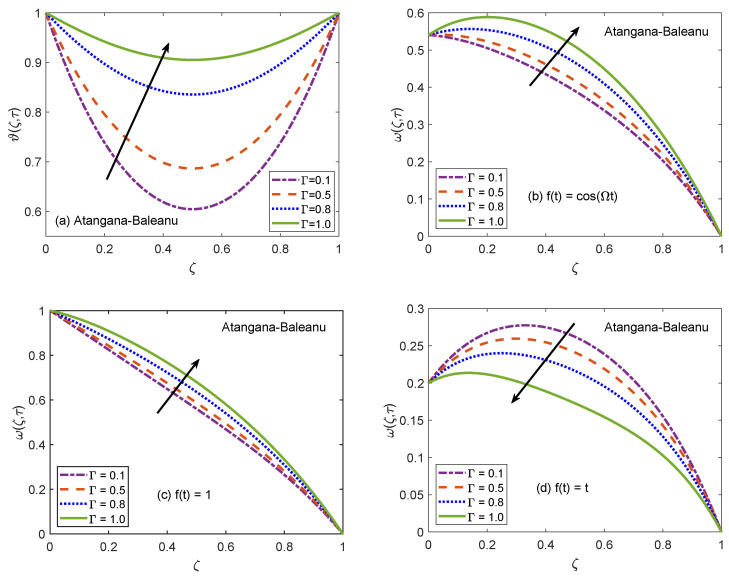
Temperature and velocity patterns of AB model for variation of Γ.

**Figure 7:**
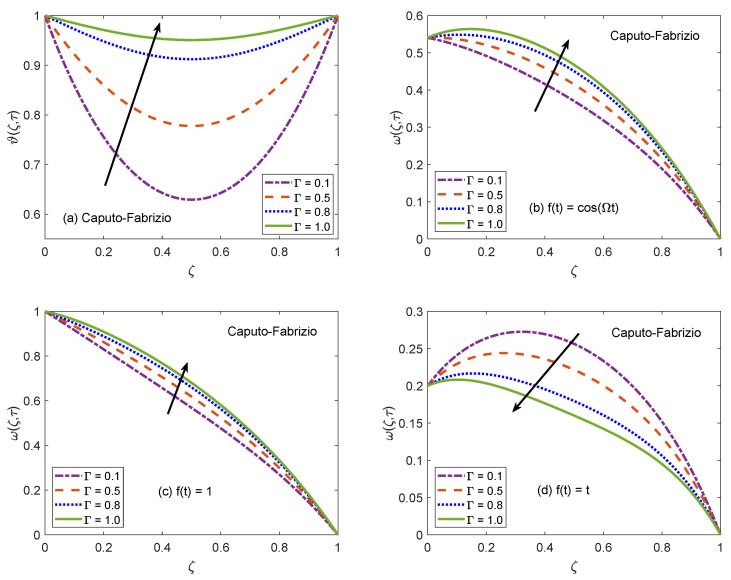
Temperature and velocity patterns of CF model for variation of Γ.

**Figure 8:**
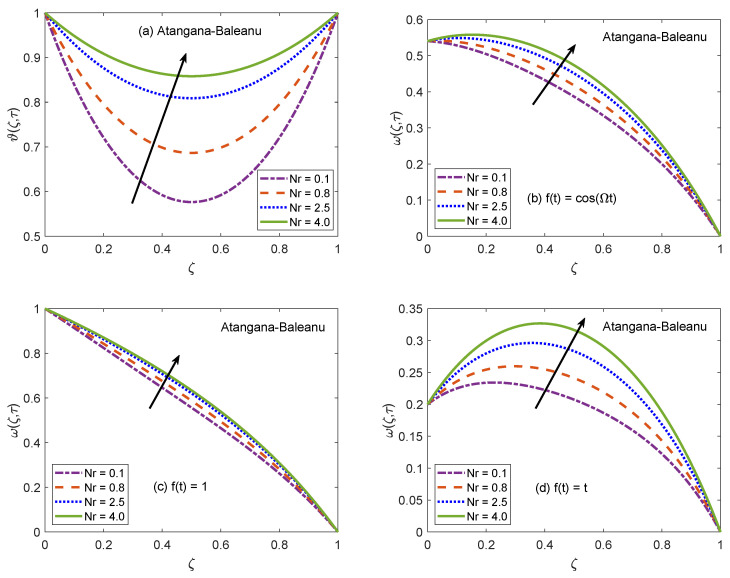
Temperature and velocity patterns of AB model for variation of Nr.

**Figure 9:**
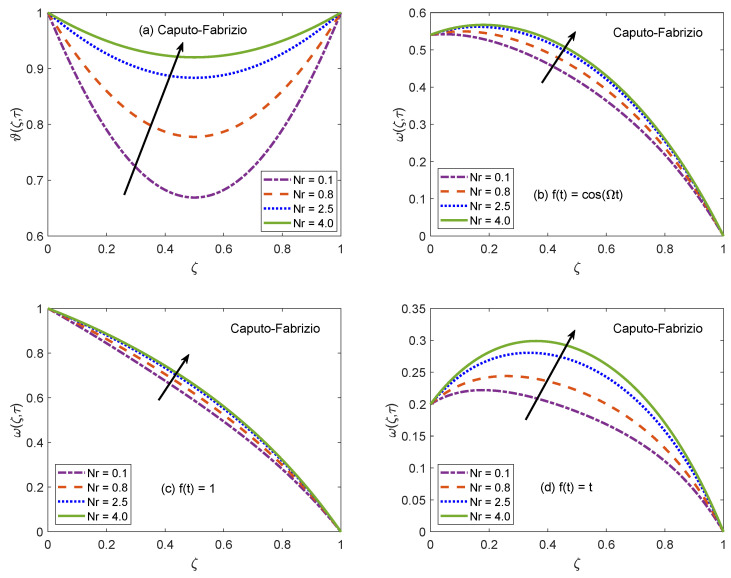
Temperature and velocity patterns of CF model for variation of Nr.

The suitability of fractional models for describing the practical physical processes is discussed with the assistance of Figure 6 and Figure 7. These figures portray the profiles of temperature and velocity distributions for multiple values of non-integer order/fractional parameter (Γ). It is spotted that Γ tends to raise the temperature profile of nanofluid and maximum value of temperature function is witnessed for Γ=1.0, which is the classical model. Likewise, velocity is also an increasing function of Γ for the cases of oscillatory and constant motion of the left channel wall. However, for the single acceleration flow condition, the behavior of velocity function is completely opposite and flow curve follows a dropping trend. This inverse behavior is primarily caused by the time difference. For single acceleration condition, the value of time is τ=0.2 and for other presented cases, this value is τ=2.0. In terms of physical argument, the momentum boundary layer gets attenuated for small value of time, which leads to declining the flow profile when the motion of wall is directly varied with time. But, corresponding to the time advancement, the boundary layer expands and a speedy flow of nanofluid is observed. Besides, the flow and energy functions execute similar trends for both AB and CF models. Here, it is significant to emphasize memory capturing and self-similar properties of fractional operators. Figure 6 and Figure 7 suggest that as compared to conventional models, the dynamics of intricate processes are effectively described by fractional models because a fractional derivative is a useful tool to capture the memory effects. In addition, it is observed that a small modification in fractional-order leads to a significant variation in the results of the fractional model thus, an adequate agreement between theoretical calculations and experimental data can be established by appropriately adjusting the order of fractional derivative. Based on the aforementioned observations, it can be concluded that using fractional derivatives for modeling the flow problems is an efficient strategy to precisely anticipate the rheology and material properties of nanofluids. The participation of radiative heat flux for the development of momentum and thermal boundary layers is of vital importance. The consequences of incorporating the radiation phenomenon in the considered model are elucidated with the support of Figure 8 and Figure 9. It is described that the energy function reveals a notable rise for elevating values of radiation parameter Nr. From the physical frame of reference, a weak thermal radiation causes the channel to drop the temperature of nanofluid because the gradient of temperature at the solid-nanofluid interface is not able to manage the sufficient heat transfer rate. As a result, the current temperature level is not maintained and the channel begins to release the energy. On the contrary, the thermal curve accepts an elevation as Nr attains higher values. For this situation, a substantial escalation in the value of Rosseland absorption coefficient ($k_{1}$) takes place subject to constant magnitudes of ${\overset{ˇ}{k}}_{nf}$ and ${⊤}_{\infty }^{*3}$. Due to this escalation, the radiative heat flux ($\frac{\partial q_{r}}{\partial \zeta^{*}}$) exhibits a dominant role and the process of heat flow through radiation occurs at a relatively quicker rate. As a consequence, the energy level of nanofluid particles is boosted and the curve associated with temperature distribution is escalated. The thermal curves in Figure 8a and Figure 9a are perfect graphical illustrations of this explanation. The graphs of velocity solutions depict that the transportation process is also influenced by thermal radiation. For three different velocity conditions, the flow of nanofluid is shown to be upsurged as the magnitude of Nr increases. The extra amount of heat transferred to nanofluid as a result of a strong radiation phenomenon is responsible for this outcome. The molecules rapidly collide each other because of their higher kinetic energy and these collisions minimize the effectiveness of coherent attraction. The ability of nanofluid to withstand flow favoring forces tends to be minor as a consequence of this physical mechanism. Thus, the flow of nanofluid is accelerated. It is clear form the plots that variation in temperature profile due to alteration of Nr is more significant than the variation in the velocity profiles because the parameter Nr primarily appears in the heat equation and partial coupling of velocity and heat equations transfers it to velocity solution as well.

**Figure 10:**
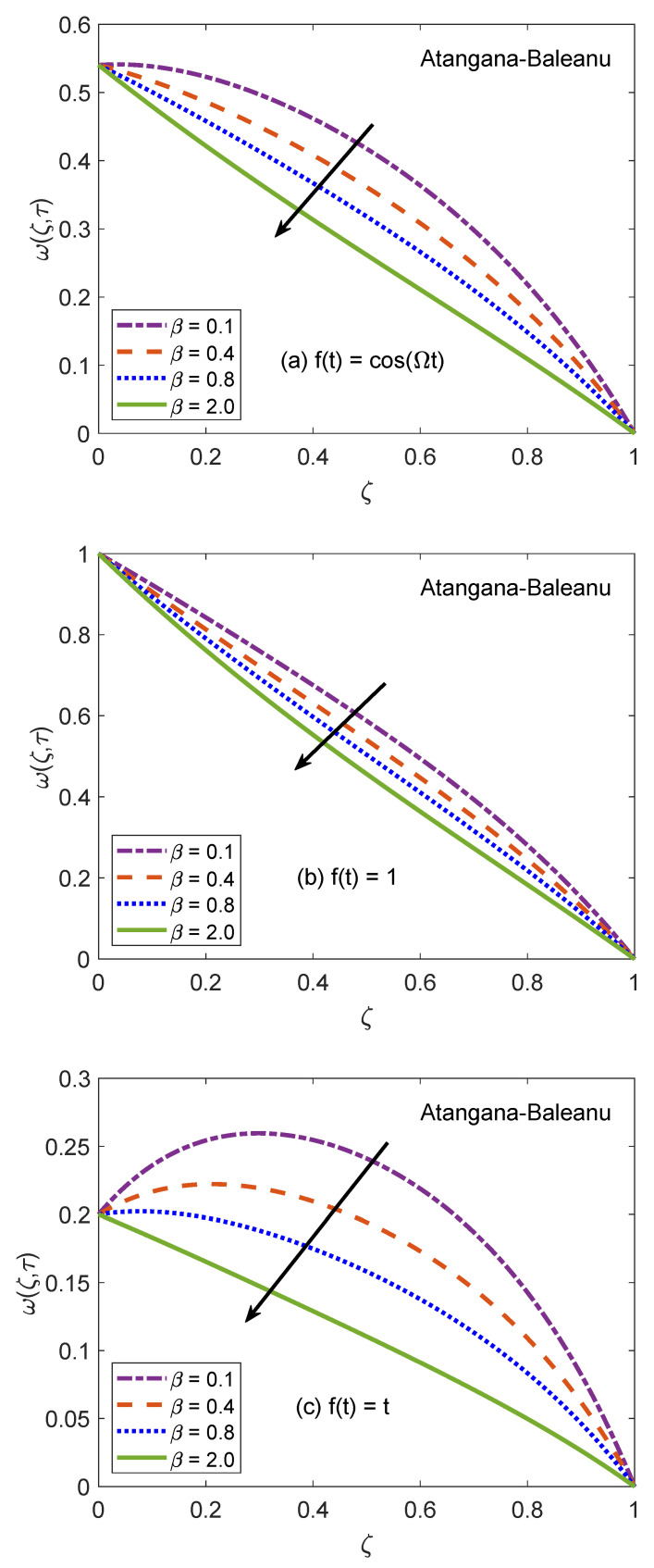
Velocity patterns for variation of β.

**Figure 11:**
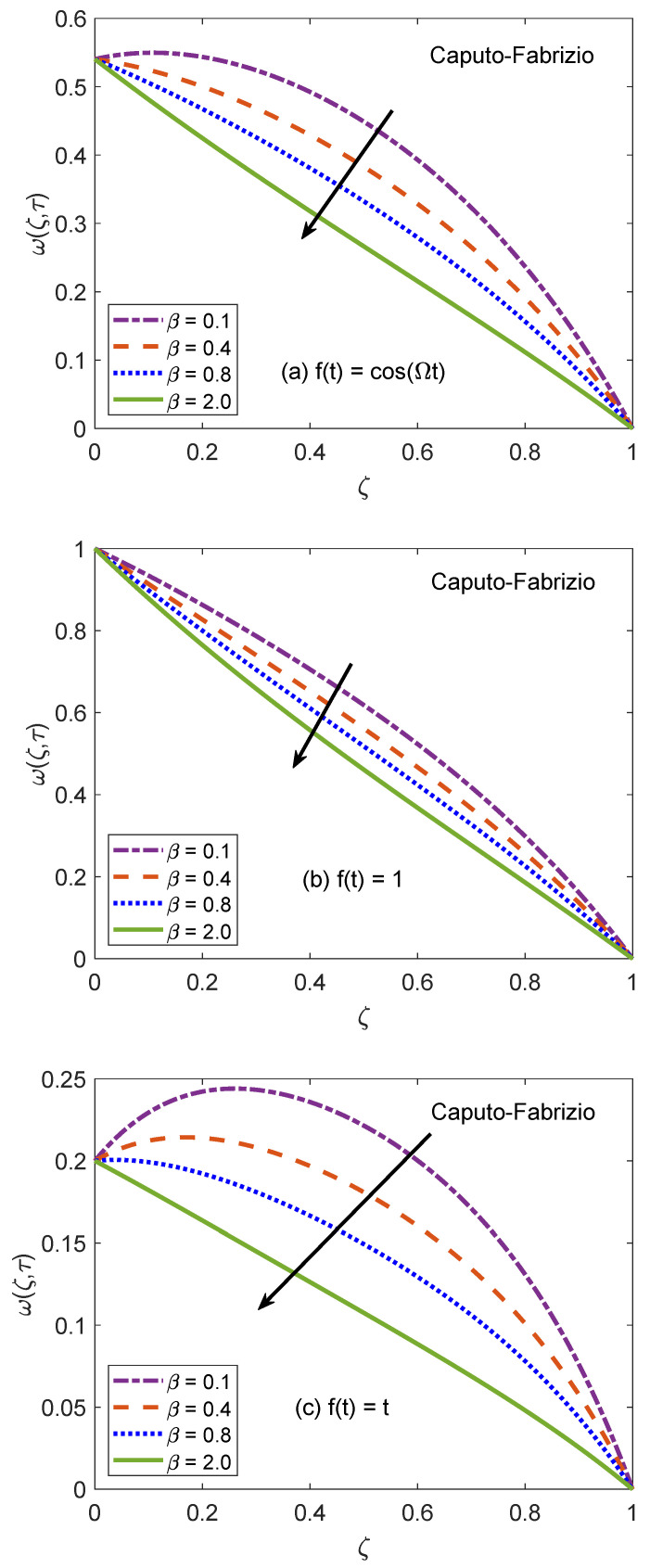
Velocity patterns for variation of β.

**Figure 12:**
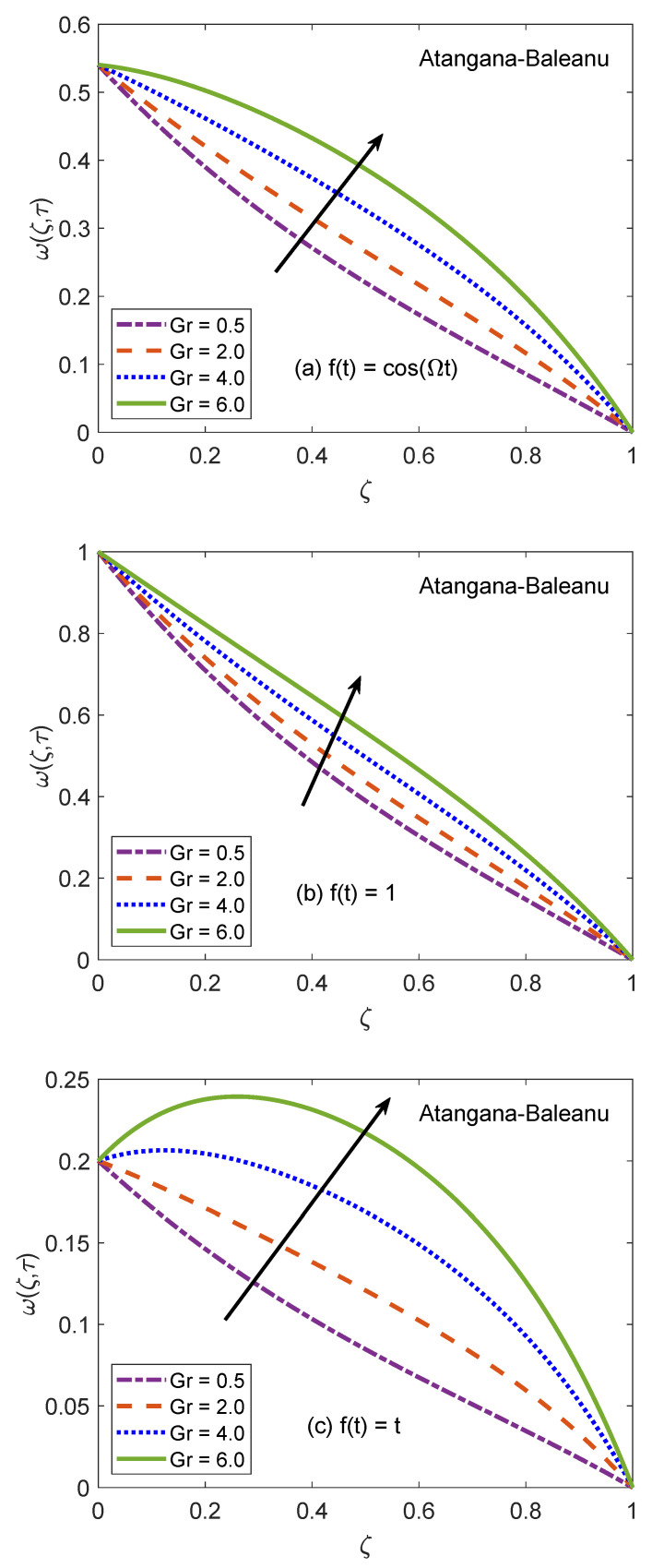
Velocity patterns for variation of Gr.

**Figure 13:**
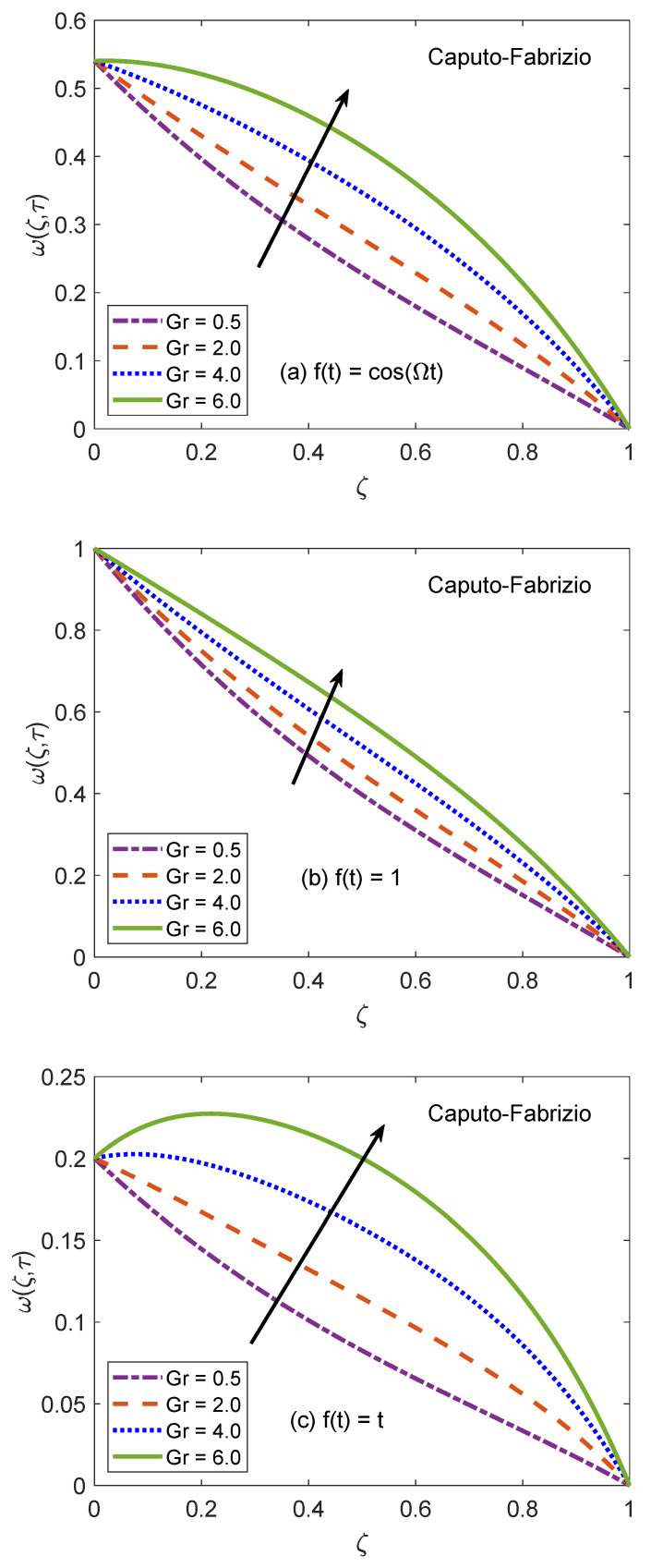
Velocity patterns for variation of Gr.

The behavior of velocity patters corresponding to the variation of Casson parameter (β) for AB and CF models is portrayed in Figure 10 and Figure 11. In the physical sense, parameter β explains the plasticity nature of Casson-type nanofluids. It is remarked that the flow of nanofluid is decelerated for growing magnitudes of β because the plasticity effects become dominant when β increases and cause an attenuation in the boundary layer. Moreover, corresponding to a larger value of β, the considered nanofluid becomes thicker and its ability to resist the deformation enhances significantly. As a consequence, the viscous effects suppress the influence of flow-favoring factors and a decline in the velocity of nanofluid takes place. Resultantly, the flow function exhibits a decay in the corresponding curve. It is essential to state that non-Newtonian features of fluid disappear for a sufficiently large magnitude of β and it acts as a Newtonian fluid. This finding also discloses that the velocity boundary layer of Casson fluid is thicker as equated to that of viscous fluid. Furthermore, the consequences of varying the value of β are not uniform for three different flow conditions. Figure 12 and Figure 13 are featured to characterize the contribution of Grashof number Gr in the establishment of flow patterns. For the flows involving free convection, Gr is studied to quantify the effects of viscous and buoyancy forces. An increment in Gr specifies supplementary heating of the channel, which leads to several changes in the flow region. For instance, there is a considerable variation in particular weights of neighboring layers of nanofluid. Moreover, it eventuates a change in the density and reduction of internal friction. The figures report an improvement in the speed of nanofluid subject to enhanced magnitude of Gr. From a physical viewpoint, the larger value of Gr induces a substantial difference in the density and convection current arises in the flow region as a consequence. The occurrence of these currents specify that the buoyancy force is established and strengthened. Meanwhile, viscous forces have insufficient strength as compared to buoyancy force and they are unable to effectively control the surge. Hence, the flow of nanofluid is accelerated and respective figures communicate uplifted profiles.

**Figure 14:**
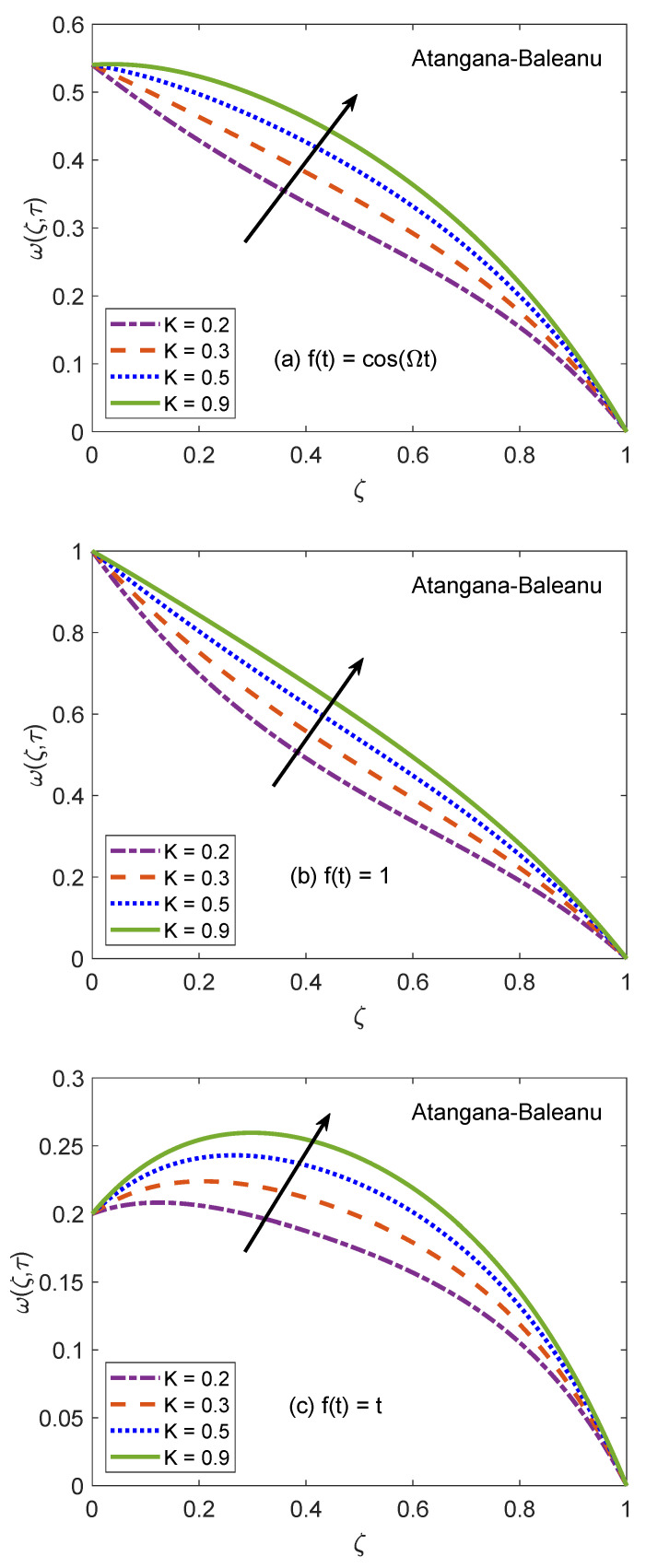
Velocity patterns of AB model for variation of *K*.

**Figure 15:**
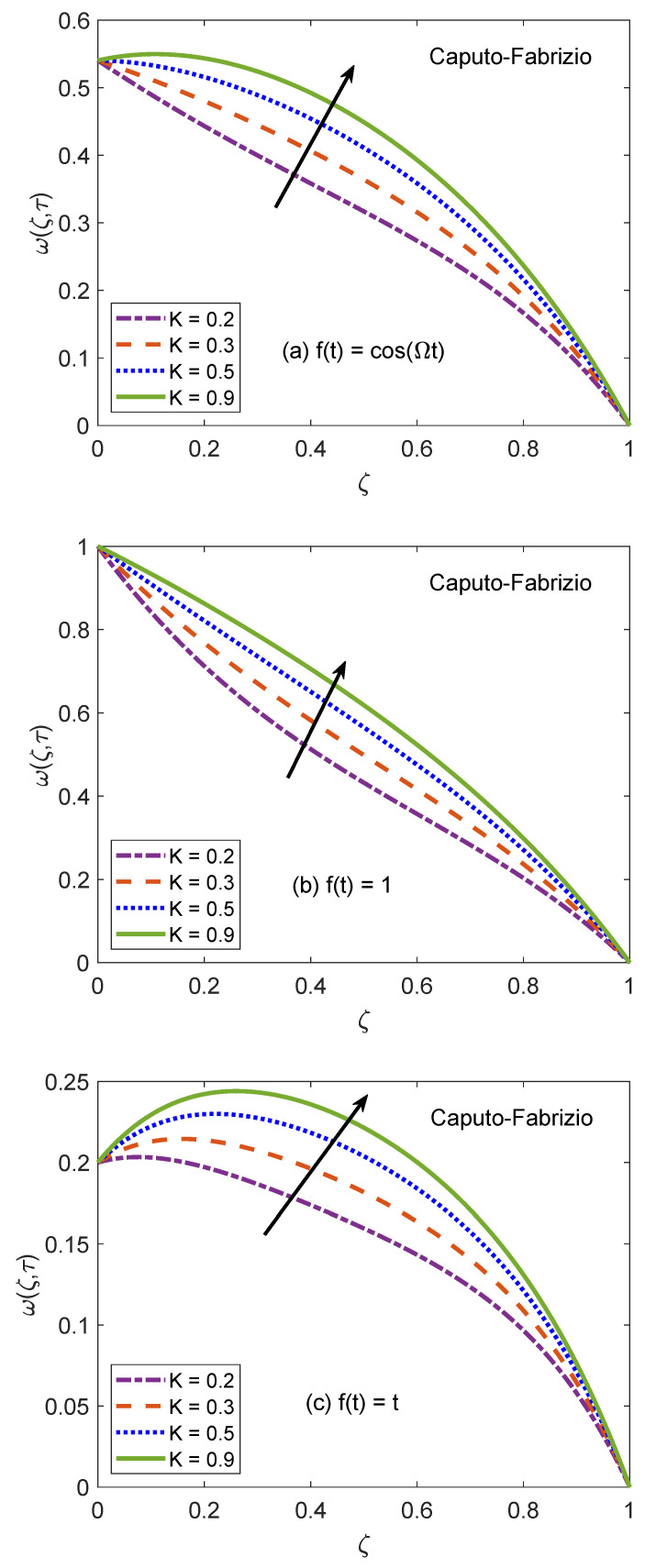
Velocity patterns of CF model for variation of *K*.

**Figure 16:**
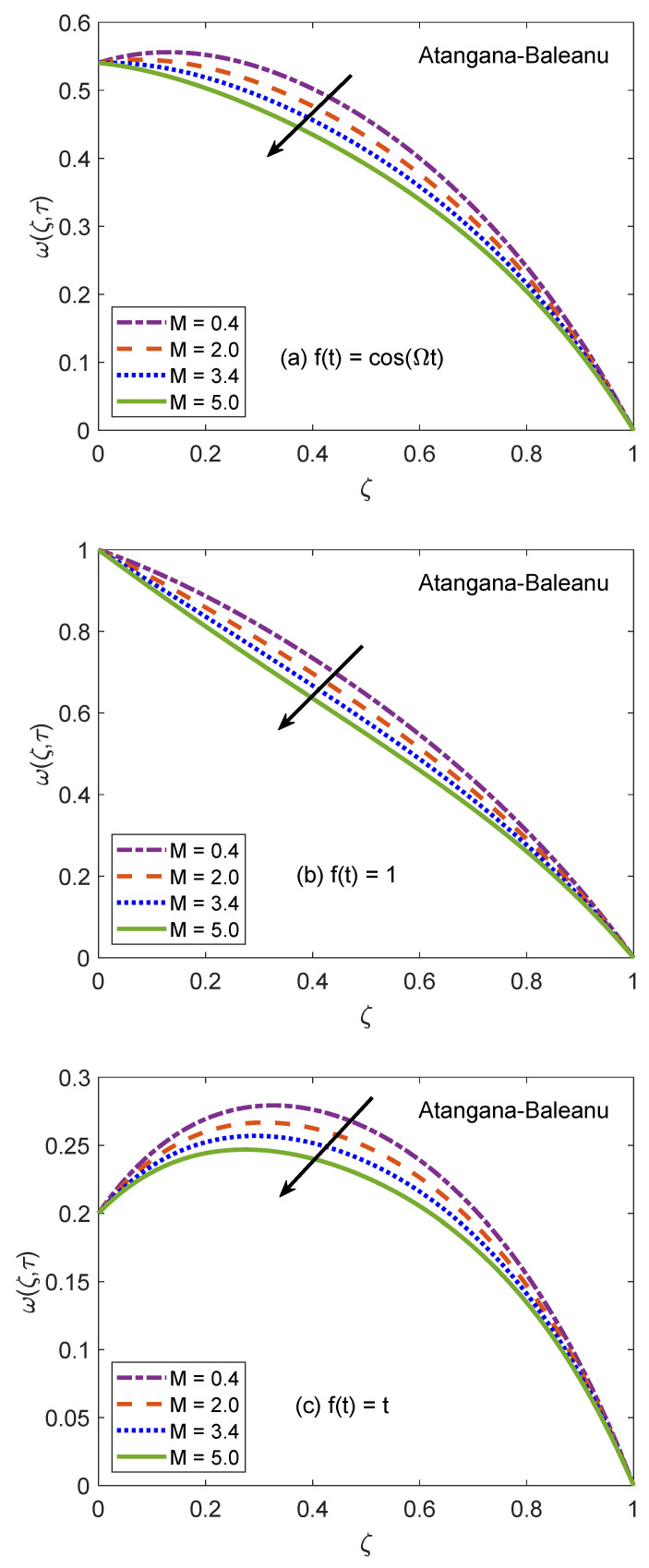
Velocity patterns of AB model for variation of *M*.

**Figure 17:**
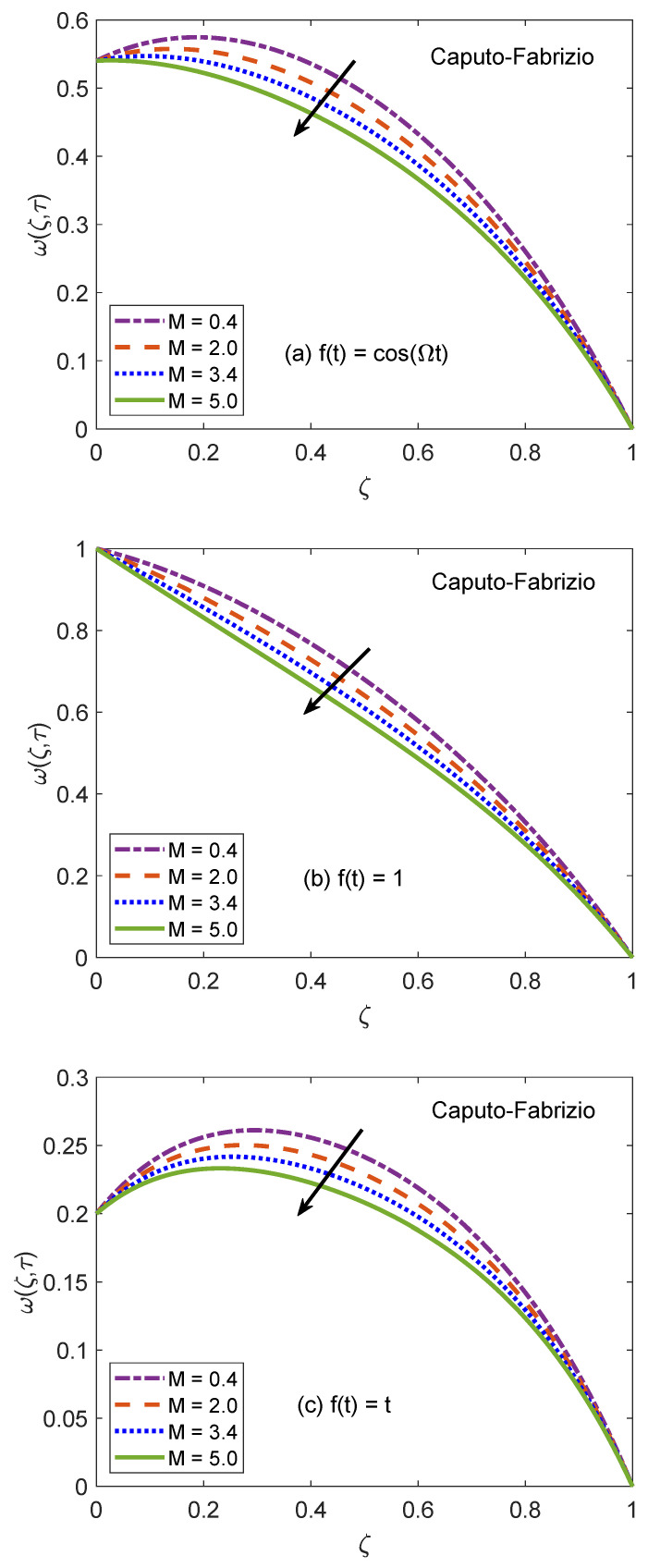
Velocity patterns of CF model for variation of *M*.

**Figure 18:**
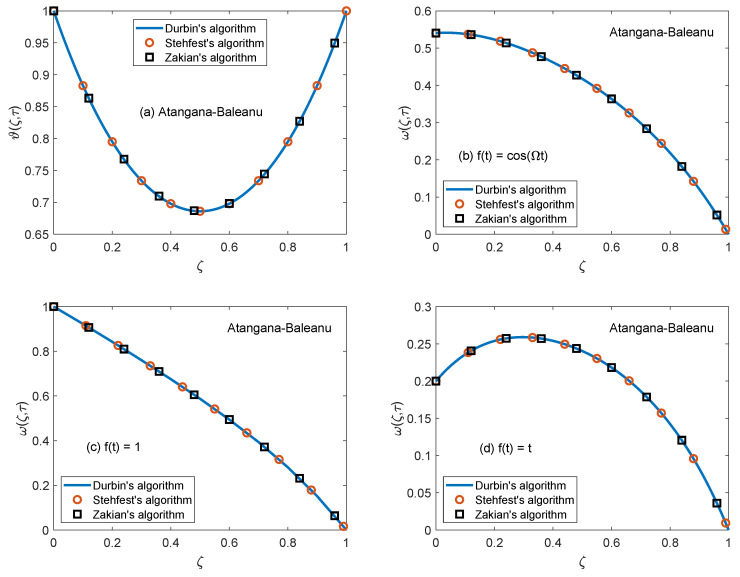
Validation of temperature and velocity fields developed through AB model.

**Figure 19:**
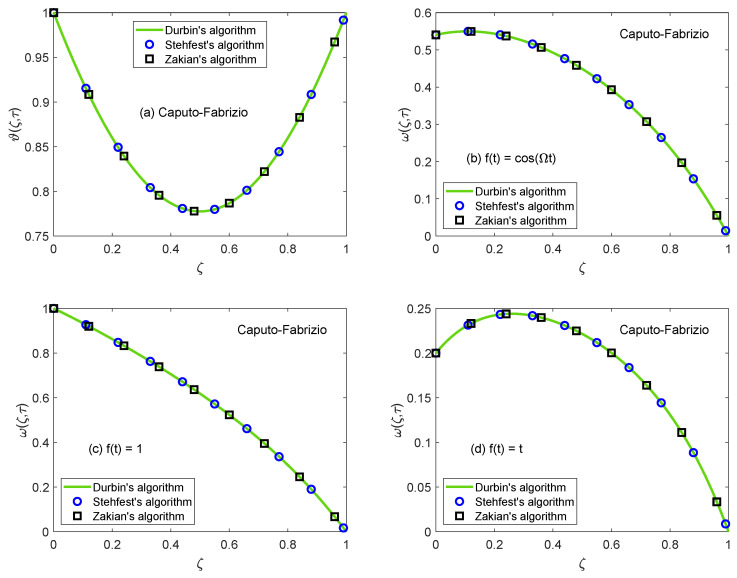
Validation of temperature and velocity fields developed through CF model.

Figure 14 and Figure 15 are produced to highlight the effectiveness of nesting the channel in a permeable medium. This analysis is conducted by modifying the porosity parameter (*K*) while all other quantities remain constant. It is discerned that as the porosity of the medium increases, the flow of nanofluid takes place at higher velocity. The preceding outcome is the result of a number of causes. The primary one is the development of momentum regime because of insignificant resistive influence of nanofluid. Secondly, the capacity of a porous material to carry forward the considered nanofluid is improved due to augmented diameter of porous holes. A drastic dwindling in the strength of dragging force is another reason for observed behavior of velocity function w(ζ,τ). The impacts of porosity parameter on flow profile are identical for both fractional models. In this modern era of the development, efficiency and functionality of various industrial devices such as MHD pumps, generators, motors, and sensors depend on the interaction between the magnetic field and electrically conducting nanofluids. Therefore, it is necessary to conduct comprehensive studies on MHD flows to evaluate various aspects of such flows. A fundamental problem in this respect is the inspection of magnetic effects on boundary layers formed on different moving and static fluid conduits, for example, cylinders or channels. Thus, the role of a magnetic field in determining the behavior of boundary layer is investigated for the present problem and outcomes are imparted through Figure 16 and Figure 17. It is perceived that the boundary layer shrinks and the motion of nanofluid retards when the dominance of magnetic field enhances. In this work, the strength of the magnetic field is symbolized with magnetic parameter *M*. The influence of Lorentz force on velocity distribution of nanofluid is the primary physical explanation for this decrease in flow velocity. The Lorentz force, generally considered as a force of resitive nature is induced due to the occurrence of the intense magnetic impacts. Due to this force, nanofluid encounters dragging effects in an anti-flow direction and experiences much significant resistance. The dropping profile of the velocity function in Figure 16 and Figure 17 for different flow situations endorse the aforementioned physical arguments. It is visualized in Figure 18 that the AB operator based fractional temperature and velocity field solutions developed through Zakian’s algorithm show a complete accordance with those anticipated through Durbin’s and Stehfest’s algorithms. Figure 19 reveals that the same observation is also true for the CF operator based solutions. Hence, it can be concluded that solutions are authentic and reliable.

For investigations incorporating energy transfer during the transportation of nanofluids inside a specific geometrical framework, Nusselt number (Nu) and skin friction coefficient ($C_{f}$) are two important quantities of physical interest to analyze the rate of heat transfer and shear stress. Table 3 is supplied to compare numerical outcomes of the aforementioned quantities for AB and CF fractional models. This table is prepared for nine values of fractional parameter (Γ) ranging from 0.1 to 0.9. Furthermore, mathematical results for $C_{f}$ are also compared for three different flow situations to observe the dominance of shear stress. Nu and $C_{f}$ exhibit totally opposite behavior subject to progression of Γ. More precisely, the value of Nu keeps reducing as Γ enhances and the maximum value occurs for Γ=0.1. On the other side, $C_{f}$ indicates an increasing trend for growing magnitude of Γ and the least value appears for Γ=0.1. In addition, the strength of shear stress is weak for single acceleration motion of the left wall as compared to those of constant and periodic motion of the wall respectively. For both fractional models, the behavior of Nu and $C_{f}$ is identical, however, the AB model produces greater values of Nu and lower values of $C_{f}$. Based on this finding, it can be remarked that the dynamics of transport phenomenon and hear transfer is adequately explicated by the AB model. The significant variation in Nu and $C_{f}$ because of Γ describes that fractional models provide more accurate and general results such that the findings of classical models can be traced and a perfect accordance with experimental evaluations can be established through a suitable alteration of the non-integer order of fractional operators. One of the main purposes of engineering nanofluids through fusion of traditional fluids and nanoparticles is to develop thermally efficient fluids so that the aim of faster cooling of conduits can be served effectively. Table 4 is furnished to anticipate the enhancement percentage of Nusselt number. It expresses that the distribution of $MoS_{2}$ nanoparticles is highly effective to improve the thermal performance of sodium alginate. It results to augment the heat transfer rate up to 9.5%, which shows that the considered nanofluid is more efficient than pure base fluid to achieve the purpose of rapid cooling. In Table 5, the numerical values of Nu for various shapes of nanoparticles are compared under the variation of volume concentration (ϕ). It is noticed that the tendency of blade shaped particles to augment the heat transfer rate is greater than platelet, cylinder, and brick shaped particles respectively. Thus, the maximum outcome of Nu against every value of ϕ is perceived for blade shape. On the basis of experimental work, Hamilton and Crosser [44] also communicated that various shapes of nanoparticles influence the heat transfer rate and thermal conductance of conventional fluids and produce a significant improvement in these characteristics. One of the major challenges of such studies is the minimization of shear stress. There occurs several technical complications when fluids are operated in industrial processes if the dominance of shear stress is significant. For example, the pumping of fluid requires more power. Thus, the use of practical techniques to control the shear stress is a principal task. For the under observation problem, efficacy of the AB model is higher than the CF model to accomplish this objective. Furthermore, a parametric study in this regard is carried out with the support of Table 6. The respective table suggests that the interaction of nanofluid with a much stronger magnetic field leads to reducing the shear stress. The decreasing value of $C_{f}$ for higher concentration of nanoparticles indicates that replacing conventional fluids with nanofluids for industrial applications is another useful technique to limit the shear stress. Besides this, lower magnitudes of parameters β, *K*, and Gr provide the desired results.

## 5. Conclusions

The major purpose of this article is to analyze the thermal conduct of a Casson-type nanofluid, flowing inside a channel, which observes the influence of permeable media. The consequences of nanoparticles’ shape factor on heat transfer during MHD convective flow of NaAlg based nanofluid are investigated for three different flow situations. The computational analysis is conducted by modeling the problem in terms of Atangana-Baleanu and Caputo-Fabrizio fractional operators. The governing equations of these models are transformed to the Laplace domain first, then Zakian’s reversal technique is employed to obtain the semi-analytic solutions. A graphical comparison is performed to evaluate that which fractional operator is more adequate and explains the observed phenomena effectively. To emphasize the importance of flow conditions, solutions for oscillatory, constant, and time-dependent boundary conditions are presented graphically. In addition, the role of other associated physical mechanisms is discussed with suitable physical justification. The outcomes of numerical simulations of skin friction coefficient and Nusselt number are expressed in tabular forms to analyze the behavior of shear stress and heat transportation ability of the considered nanofluid. Some primary findings of this analysis are summarized as

- Molybdenum-disulfide nanoparticles produce a 9.5% enhancement in the thermal efficiency of sodium alginate, which enhances its usefulness for practical applications.
- Nanofluid flows with maximum velocity when the wall exhibits a constant motion.
- The Lorentz force appearing because of magnetic effects tends to retard the flow of nanofluid.
- The fractional model formulated in the form of the Atangana-Baleanu operator is more effective than the model prepared by applying the Caputo-Fabrizio operator.
- The fractional model outperforms the usual model in terms of achieving goals of diminished skin friction and augmented heat transfer rate.
- Suspending $MoS_{2}$ nanoparticles of blade shapes causes maximum improvement in thermal features of the base fluid. However, the percentage enhancement in heat transfer rate due to the distribution of cylindrical, brick, and platelet shapes is 5.4%, 3.9%, and 6.5% respectively.
- The existence of a dominant magnetic field and a greater concentration of nanoparticles in the base fluid are favorable factors to restrict skin friction.
- The lowest skin friction is observed when the motion of the left wall directly depends on time.
- The fractional parameter produces an elevation in the profile of the temperature function.
- The augmented thermal properties and lubrication features of the $MoS_{2}$ nanofluid signify its role in mechanical applications.
- The porous medium and radiative flux adequately contribute to the development of boundary layer flow.

## 6. Future Research Work

- This problem can be extended for different kinds of hybrid nanofluids.
- This model can be used for other geometrical configurations.
- This work can be modified for two and three-dimensional problems.

## Figures and Tables

**Table 1:** Numerical values of $\overset{ˇ}{a}$, $\overset{ˇ}{b}$, and ψ for considered shapes [29].

Model	Blade	Platelet	Cylinder	Brick
$\overset{ˇ}{a}$	123.3	612.6	909.4	471.4
$\overset{ˇ}{b}$	14.6	37.1	13.5	1.9
ψ	0.36	0.52	0.62	0.81

**Table 2:** Values for thermo-physical quantities of NaAlg and ${MoS}_{2}$ [29,46].

Quantities	Carrier Fluid	Nanoparticles
	NaAlg	${\mathrm{MoS}}_{2}$
${\overset{ˇ}{\beta }}_{1}\times 10^{-5}$	0.99	2.84
$\overset{ˇ}{C_{p}}$	4175	397.21
$\overset{ˇ}{\rho }$	989	5.06 × 10${}^{3}$
$\overset{ˇ}{k}$	0.6376	904.4
$\overset{ˇ}{\sigma }\times 10^{-4}$	2.6	2.09

**Table 3:** Comparative numerical analysis of $C_{f}$ and Nu for fractional parameter Γ.

Γ	Atangana-Baleanu				Caputo-Fabrizio			
	$C_{f}$			Nu	$C_{f}$			Nu
	f(t)=cos(Ωt)	f(t)=1	f(t)=t		f(t)=cos(Ωt)	f(t)=1	f(t)=t	
0.1	−1.2508	−2.5543	−7.7980	2.7619	−1.1688	−2.4604	−7.7295	2.5794
0.2	−1.2180	−2.5161	−7.7665	2.6876	−1.0627	−2.3379	−7.6356	2.3420
0.3	−1.1635	−2.4526	−7.7140	2.5641	−0.9437	−2.1996	−7.5252	2.0749
0.4	−1.0864	−2.3626	−7.6390	2.3891	−0.8136	−2.0475	−7.3974	1.7816
0.5	−0.9849	−2.2439	−7.5380	2.1589	−0.6769	−1.8862	−7.2523	1.4715
0.6	−0.8577	−2.0943	−7.4058	1.8692	−0.5416	−1.7246	−7.0925	1.1624
0.7	−0.7051	−1.9134	−7.2365	1.5201	−0.4192	−1.5757	−6.9256	0.8793
0.8	−0.5327	−1.7064	−7.0260	1.1223	−0.3186	−1.4500	−6.7647	0.6419
0.9	−0.3518	−1.4852	−6.7788	0.6980	−0.2392	−1.3480	−6.6252	0.4437

**Table 4:** Numerical analysis of percentage enhancement in Nusselt number due to volume fraction of nanoparticles.

ϕ	Γ	τ	Nr	Nu	Enhancement %
0.00	0.5	2.0	0.2	2.0274	-
0.01	0.5	2.0	0.2	2.0829	2.7375
0.02	0.5	2.0	0.2	2.1331	5.2136
0.03	0.5	2.0	0.2	2.1786	7.4578
0.04	0.5	2.0	0.2	2.2199	9.4949

**Table 5:** Numerical analysis of Nusselt number enhancement for different shapes of nanoparticles.

ϕ	Nu	Nu	Nu	Nu
	Brick	Blade	Cylinder	Platelet
0.00	2.0274	2.0274	2.0274	2.0274
0.01	2.0488	2.0829	2.0571	2.0638
0.02	2.0691	2.1331	2.0851	2.0978
0.03	2.0885	2.1786	2.1114	2.1294
0.04	2.1069	2.2199	2.1361	2.1589

**Table 6:** Numerical analysis of associated parameter’s impact on skin friction.

*K*	Gr	β	ϕ	*M*	$C_{f}$		
0.9	7.0	2.0	0.02	3.0	f(t)=cos(Ωt)	f(t)=1	f(t)=t
0.3	-	-	-	-	−1.3887	−4.2225	−11.1185
0.5	-	-	-	-	−0.7267	−3.0890	−9.0613
0.9	-	-	-	-	−0.2333	−2.2439	−7.5380
-	2.0	-	-	-	−2.8273	−4.3216	−9.6157
-	4.0	-	-	-	−2.0904	−3.4905	−8.7846
-	6.0	-	-	-	−1.3534	−2.6595	−7.9535
-	-	0.4	-	-	−4.6609	−7.5958	−18.3955
-	-	0.8	-	-	−2.3391	−4.2315	−11.6004
-	-	2.0	-	-	−0.9849	−2.2439	−7.5380
-	-	-	0.02	-	−0.9849	−2.2439	−7.5380
-	-	-	0.03	-	−1.6940	−3.3523	−10.1517
-	-	-	0.04	-	−2.6144	−4.7440	−13.3267
-	-	-	-	2.0	−0.4537	−1.9039	−6.9275
-	-	-	-	3.4	−0.7084	−2.3763	−7.7760
-	-	-	-	5.0	−0.7261	−2.8866	−8.6957

## Sample Availability

Samples of the compounds are available from the authors.
